# Supplementary material for: Dual targeted extracellular vesicles regulate oncogenic genes in advanced pancreatic cancer
Source: Nat Commun. 2023 Oct 23;14:6692. doi: 10.1038/s41467-023-42402-3 (PMC10593751; doi:10.1038/s41467-023-42402-3)
Supplement: Supplementary file 1 — Supplementary Information [file 41467_2023_42402_MOESM1_ESM.pdf]

Supplementary information

Dual targeted extracellular vesicles regulate oncogenic genes in advanced pancreatic cancer.

Supplementary Notes

Supplementary Note 1 | Two-dimensional (2D) TACE modeling of transmembrane membrane potential (TMP).

Supplementary Tables

Supplementary Table 1 | Canonical pathway analysis for EV transcriptome from TACE stimulated hBMSCs with or without TP53 plasmid.
Supplementary Table 2 | Canonical pathway analysis for EV transcriptome from TACE stimulated MEFs with or without TP53 plasmid.
Supplementary Table 3 | List of sequence of siRNA used in dtEV studies**.**

Supplementary Table 4 | TACE simulation parameters used in modeling.

Supplementary Table 5 | List of the sequence of probes used in FISH staining.

Supplementary Table 6 | The sequence and specificity of the probes used for ILN assay.

Supplementary Table 7 | Changes of major microRNAs in EVs from TACE stimulated hBMSCs with PBS or TP53 plasmid.

Supplementary Figures

Supplementary Figure 1 | TACE enables more efficient cellular CD64 expression compared to conventional bulk electroporation.
Supplementary Figure 2 | Quantification of cargoes in dtEV by using calibration curve with standard rtCD64 and synthetic RNAs.
Supplementary Figure 3 | Dynamics of the dtEV production by sequential TACE (sTACE) for donor cell BMSC.
Supplementary Figure 4 | The sequence and specificity of the probes and antibodies used for staining of FISH and immunofluorescence in donor cells.
Supplementary Figure 5 | Single dtEV characterization using immune lipoplex nanoparticle (ILN)-Total Internal Reflection Fluorescence Microscopy (TIRFM) assay.
Supplementary Figure 6 | The sequence and specificity of the probes used for ILN assay.

Supplementary Figure 7 | Changes of transcripts in collected EVs from TACE stimulated hBMSCs with TP53 plasmid.

Supplementary Figure 8 | Changes of major microRNAs in EVs from TACE stimulated hBMSCs with PBS or TP53 plasmid

Supplementary Figure 9 | Changes of transcripts in collected EVs from TACE stimulated MEFs with TP53 plasmid.

Supplementary Figure 10 | Stability of hmAbs on EVs and penetration comparison of different nanocarriers in PANC-1 spheroids

Supplementary Figure 11 | Dynamics of single-EV analysis reveals dtEVs could accelerate endocytosis in PANC-1 cells.

Supplementary Figure 12 | Three-dimensional (3D) quantification of single cells reveals enhanced dtEV accumulation in living PANC-1 cells.

Supplementary Figure 13 | Targeting and internalization ability of dtEVs with αEGF or αROR1 in PANC-1 spheroid.

Supplementary Figure 14 | Design of the anti-GFP_mCherry fluobody.

Supplementary Figure 15 | *In vitro* therapeutic efficacy results of various EVs in PANC-1 cells.

Supplementary Figure 16 | Cell cycle analysis by PI staining in PANC-1 cells with dtEV treatment.

Supplementary Figure 17 | Therapeutic effects of dtEVs on viability of PANC-1 cells

Supplementary Figure 18 | The SA-β-galactosidase (SA-β-gal) activity assay showed dtEV-induced OIS.

Supplementary Figure 19 | dtEVs suppressed KRAS expression in tumours from orthotopic PANC-1 mouse model.

Supplementary Figure 20 | Toxicity and blood circulation of dtEVs *in vivo*

Supplementary Figure 21 | dtEVs suppressed KRAS expression in PDAC tumours from PDX mice.

Supplementary Figure 22 | Histological analysis by hematoxylin and eosin (H&E) staining of tumour and main organs from subcutaneous PDX mice.

Supplementary Figure 23 | Pathological analysis for the tissue sections from dtEV-treated subcutaneous (subQ) PDX mice.

Supplementary Figure 24 | Analysis of combinative gene therapy against intrinsic chemoresistance of PANC-1 cells to Gemcitabine (GEM).

Supplementary Figure 25 | Tumour size measurements by positron emission tomography and computed tomography (PET-CT) in orthotopic PDX PDAC mice.

Supplementary Figure 26 | The DNase treatment is essential for removal of the plasmid DNA contamination in dtEV samples.

Supplementary Figure 27 | Comparison of RNA loading in dtEVs collected from TEI isolation reagent and TFF system.

Supplementary Figure 28 | Comparison of dtEV measurement between DLS and NTA.

Supplementary Figure 29 | dtEVs with an integrated membrane exhibit resistance to degradation.

Supplementary Figure 30 | Collagen sealing in transcytosis assay prevented the leakage of bypassed dtEVs.

Supplementary Figure 31 | Uncropped blots.

Supplementary References

Supplementary Note 1 | Two-dimensional (2D) TACE modeling of transmembrane membrane potential (TMP). A simplified 2D model is built for finite-element analysis (FEA). COMSOL Multiphysics 5.5 with MATLAB is used to analyze the stationary transmembrane membrane potential (TMP) when the cell is subjected to an external electric field. The electric potential distribution and field intensity are obtained by solving the following governing equations:

Conservation of charge: $\nabla \cdot \left(\sigma \nabla V + \frac{\partial (\varepsilon \nabla V)}{\partial t} \right) = 0,$

Transmembrane potential: $\Delta V_m = V(\theta, R) - V(\theta, R+d)$,

where σ is electric conductivity; V is voltage; ε is relative permittivity; t is time; R is the radius of the cell; d is the membrane thickness. Model geometry and material properties are shown in **Supplementary table 4**.

Supplementary Table 1 | Canonical pathway analysis for EV transcriptome from TACE stimulated hBMSCs with or without TP53 plasmid. The darker the color, either green or red, the stronger the significance. More details can be found in GEO accession GSE223409.

		TACE		TACE with TP53 plasmid	
Category	Pathway Description	Appeared	Disappeared	Appeared	Disappeared
Metabolism	Purine metabolism	5.80E - 02			
	Fatty acid elongation	8.90E - 02			
	Steroid biosynthesis			3.50E - 02	
	N-Glycan biosynthesis			3.40E - 02	
	Metabolic pathways			8.50E - 02	
	Amino sugar and nucleotide sugar metabolism	1.60E - 02		8.30E - 02	
Genetic information Processing	mRNA surveillance pathway	1.20E - 03		2.90E - 02	
	Ribosome biogenesis in eukaryotes	6.10E - 03		2.20E - 02	
	RNA transport	1.50E - 03		1.30E - 02	
	Basal transcription factors	8.60E - 02			
	Spliceosome	1.60E - 08		9.30E - 06	
	Base excision repair	6.20E - 03			
	DNA replication	1.10E - 04		6.80E - 03	
	Mismatch repair	1.80E - 02		5.50E - 02	
	Nucleotide excision repair	1.40E - 02		2.80E - 02	
	Protein processing in endoplasmic reticulum	2.00E - 04	5.60E - 02	1.00E - 03	
	RNA degradation	3.70E - 02			
	Ubiquitin mediated proteolysis	1.20E - 02			
Cellular Processes	Cell cycle	9.40E - 05		2.10E - 03	
	Endocytosis		1.70E - 02		3.70E - 02
	Oocyte meiosis	7.80E - 02			
	p53 signaling pathway			2.60E - 02	
	Phagosome	2.90E - 02	1.70E - 03		2.80E - 02
	Regulation of actin cytoskeleton			7.70E - 02	
Immune system	Antigen processing and presentation		4.00E - 03		
	Vasopressin-regulated water reabsorption				4.50E - 02
	Protein digestion and absorption		3.90E - 02		

Supplementary Table 2 | Canonical pathway analysis for EV transcriptome from TACE stimulated MEFs with or without TP53 plasmid. The yellow highlighted ones are identical pathways enriched in both MEFs and hBMSCs. The darker the color, either green or red, the stronger the significance. More details can be found in GEO accession GSE223409.

		TACE		TACE with TP53 plasmid	
Category	Pathway Description	Appeared	Disappeared	Appeared	Disappeared
Metabolism	Biosynthesis of amino acids	8.00E - 02			
	Biosynthesis of cofactors	6.40E - 02			
	Biosynthesis of unsaturated fatty acids			9.00E - 02	
	Butanoate metabolism	1.80E - 02			
	Carbon metabolism	6.30E - 03			
	Cysteine and methionine metabolism	4.10E - 03			
	Fatty acid metabolism	8.90E - 03			
	Folate biosynthesis	1.60E - 02			
	Fructose and mannose metabolism	4.70E - 02			
	Galactose metabolism				5.30E - 02
	Glutathione metabolism	5.60E - 02			
	Metabolic pathways	8.10E - 04	3.20E - 02		2.50E - 07
	Nucleotide metabolism				3.40E - 02
	Oxidative phosphorylation	3.30E - 02			7.50E - 36
	Propanoate metabolism	2.90E - 02			
	Pyrimidine metabolism	6.10E - 02			
	Valine, leucine and isoleucine degradation	1.30E - 03			
Environmental information processing	DNA replication	1.90E - 03			
	Mismatch repair	8.80E - 03			
	mRNA surveillance pathway	1.80E - 03			
	Nucleocytoplasmic transport	1.40E - 02			
	Nucleotide excision repair	2.30E - 02			
	Proteasome	8.40E - 03			
	Protein processing in endoplasmic reticulum				2.30E - 06
	Ribosome				1.80E - 10
	Ribosome biogenesis in eukaryotes	8.10E - 02			
	RNA degradation	1.30E - 03			
	Spliceosome	2.90E - 06			1.70E - 02
	Ubiquitin mediated proteolysis	4.60E - 04			
Cellular Processes	AMPK signaling pathway	3.20E - 03			

	CGMP - PKG signaling pathway				8.70E - 02
	ECM - receptor interaction		2.10E - 02		
	FoxO signaling pathway	4.10E - 03			3.20E - 02
	HIF - 1 signaling pathway	1.00E - 04			3.10E - 02
	MAPK signaling pathway			4.70E - 02	2.30E - 02
	PI3K - Akt signaling pathway				8.20E - 02
	Sphingolipid signaling pathway	2.00E - 02			
	TGF - beta signaling pathway	7.30E - 02			
	TNF signaling pathway	3.70E - 03			
Immune system	adherent junction	1.80E - 02			
	Apoptosis				7.70E - 03
	Autophagy - animal	9.20E - 02			1.90E - 04
	Cell cycle	1.00E - 06			
	Cellular senescence	9.20E - 02			3.60E - 03
	Endocytosis	7.80E - 03			4.10E - 06
	Ferroptosis	1.70E - 02			
	Lysosome		6.10E - 02		3.90E - 02
	Mitophagy- animal				3.10E - 03
	Oocyte meiosis	2.10E - 03			9.20E - 02
	p53 signaling pathway	5.60E - 02			
	Peroxisome	5.30E - 03			
	Regulation of actin cytoskeleton				3.00E - 02
	Tight junction				7.10E - 02
	Antigen processing and presentation				4.80E - 02
	Axon guidance	4.30E - 02			
	C - type lectin receptor signaling pathway				6.30E - 02
	Cardiac muscle contraction				1.60E - 03
	Endocrine and other factor - regulated calcium reabsorption		6.20E - 02		
	Fc gamma R - mediated phagocytosis	2.50E - 02			
	IL - 17 signaling pathway	2.50E - 02			2.30E - 02
	Insulin signaling pathway	8.30E - 02			4.70E - 02
	Leukocyte transendothelial migration	8.10E - 02			
	Neurotrophin signaling pathway				9.10E - 05
	NOD - like receptor signaling pathway				9.60E - 02
	Progesterone - mediated oocyte maturation	6.10E - 02			

	Retrograde endocannabinoid signaling				5.80E - 11
	Synaptic vesicle cycle		1.40E - 02		2.10E - 03
	Thermogenesis				2.50E - 22
	Thyroid hormone signaling pathway	8.70E - 02			
	Vasopressin - regulated water reabsorption				4.50E - 02

Supplementary Table 3 | List of sequences of siRNAs used in dtEV studies.

Targets	Sequence
KRAS_wt	AACUACGCCA**C**CAGCUCCAAC
KRAS_G12D	AACUACGCCA**U**CAGCUCCAAC
KRAS_G12V	AACUACGCCA**A**CAGCUCCAAC
Scramble	CGAGGGCGACUUAACCUUAGG
GLI1	AUAAUUACACACAAGCUGAGC
hTM4SF1	AAUAUUAGCCGCGAUGCACAG
hMGMT	UAUUUCGUGCAGACCCUGCUC
hSTK33	UAAUAACAUGUACAUUACGAC
hMUC1	UUAUACUGAUUGAACUGUGUC

Supplementary Table 4 |TACE simulation parameters used in modeling.

	Parameter	Symbol	Value
Geometry	Length of cell channel	L	200 µm
	Width of cell channel	W	30 µm
	Length of poration channel	l	10 µm
	Radius of poration channel	r_2	0.1-5 µm
	Cell radius	R	7.5 µm
	Cell membrane thickness	d	5 nm
	Gap between cell and channel	d_2	10 nm
Material	Extracellular medium conductivity	σ_0	0.8 S/m
	Cytoplasm conductivity	σ_1	0.2 S/m
	Cell membrane conductivity	σ_m	5×10^{-7} S/m

Supplementary Table 5 | List of the sequence of probes used in FISH staining.

Name	GeneGlobe ID	Location	Sequence
TP53_probe1	LCD0176602-BKA	1012-1033	TCAGCTCTCGGAACATCTCGAA
TP53_probe2	LCD0176603-BKA	485-505	TGTGCTGTGACTGCTTGTAGA
TP53_probe3	LCD0176604-BKA	802-822	AACACGCACCTCAAAGCTGTT
siKRAS_probe	YCD0077975-BCA	20	TTGGAGCTGATGGCGTAGTT
Scramble_probe1 of TP53	-	-	ATCCAACCGATCCGGATGTACT
Scramble_probe2 of TP53	-	-	GTGTTACGTGTCTAGATGTCG
Scramble_probe3 fTP53	-	-	GCATTAATCACACGCCGATAC
Scramble_probe4 of siKRASG12D	-	-	GGTTCAGGTTGATTCGTGGA

Supplementary Table 6 |The sequence and specificity of the probes used for ILN assay.

Probe	Sequence
TP53 mRNA (NM_000546.6)	5'-CTC CGT /CY5 /CAT GTG CTG TGA CTT CAC AGC ACA TG -BHQ3-3'
siKRASG12D	5'-CTT GGA /CY5 /GCT GAT GGC GTA GTT ACG CCA TCA GC -BHQ3-3'

Supplementary Table 7 | Changes of major microRNAs in EVs from TACE stimulated hBMSCs with PBS or TP53 plasmid.

Mature miR ID	BMSC_control	BMSC_plasmid	BMSC_PBS
hsa-miR-370-3p	4.36	6.64	5.38
hsa-miR-92a-1-3p	10.19	12.05	11.54
hsa-let-7d-3p	5.01	6.66	6.98
hsa-miR-7-1-5p	6.09	7.65	8.38
hsa-miR-423-3p	8.10	9.39	9.12
hsa-miR-199a-1-3p	11.39	10.84	9.37
hsa-miR-99a-5p	8.41	6.30	4.62
hsa-miR-26b-5p	9.38	7.26	7.41
hsa-miR-27b-3p	11.60	9.05	5.88
hsa-miR-23b-3p	9.62	6.94	8.03
hsa-miR-148a-3p	10.85	8.11	6.77
hsa-let-7c-5p	7.09	4.34	3.08
hsa-miR-30c-1-5p	8.42	5.22	5.77
hsa-miR-374b-5p	6.60	3.29	5.45
hsa-miR-140-5p	6.01	2.54	2.49
hsa-miR-26a-1-5p	11.69	8.14	9.10
hsa-miR-218-1-5p	5.91	2.32	4.88
hsa-miR-382-3p	6.45	2.83	4.51
hsa-miR-140-3p	9.89	6.23	6.86
hsa-miR-889-3p	5.78	2.07	4.43
hsa-miR-15b-5p	7.92	4.15	6.15
hsa-miR-654-3p	6.96	3.04	5.31
hsa-miR-30b-5p	6.68	2.47	4.08
hsa-miR-30a-3p	7.12	2.89	4.71
hsa-miR-451a-5p	7.39	2.83	2.64
hsa-miR-335-5p	7.25	1.87	2.33
hsa-miR-9-1-5p	6.40	0.00	1.14
hsa-miR-183-5p	7.77	0.62	0.00
hsa-miR-182-5p	8.71	0.34	2.44

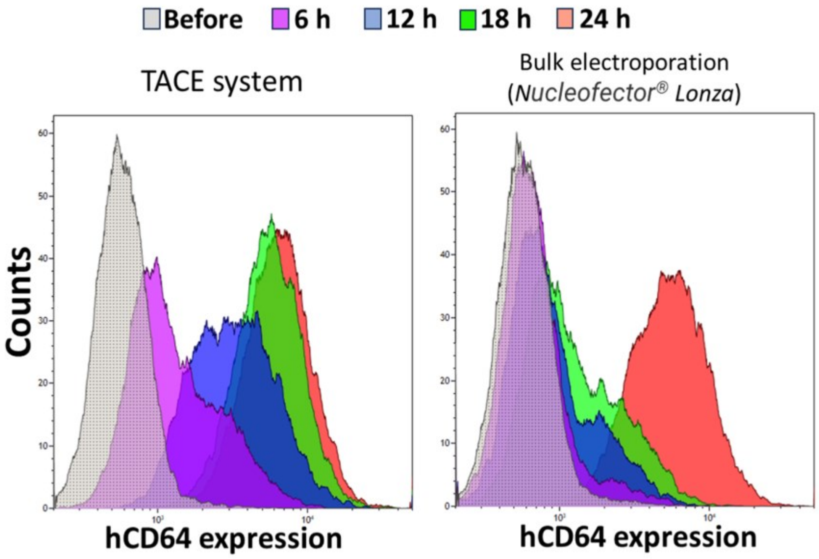

Supplementary Figure 1 | TACE enables more efficient cellular CD64 expression compared to conventional bulk electroporation. We conducted measurements of human CD64 expression in MEF cells at different time points (6, 12, 18, and 24 h) following either TACE (left) or bulk electroporation (right) transfection. By leveraging the reduced cellular damage associated with TACE, we observed that the electroporated cells exhibited better recovery and initiated CD64 expression as early as 6 h after TACE electroporation. Conversely, it took over 18 h for stable CD64 expression to be observed from plasmid DNA in cells by conventional bulk electroporation. The flow data is representative of three independent experiments.

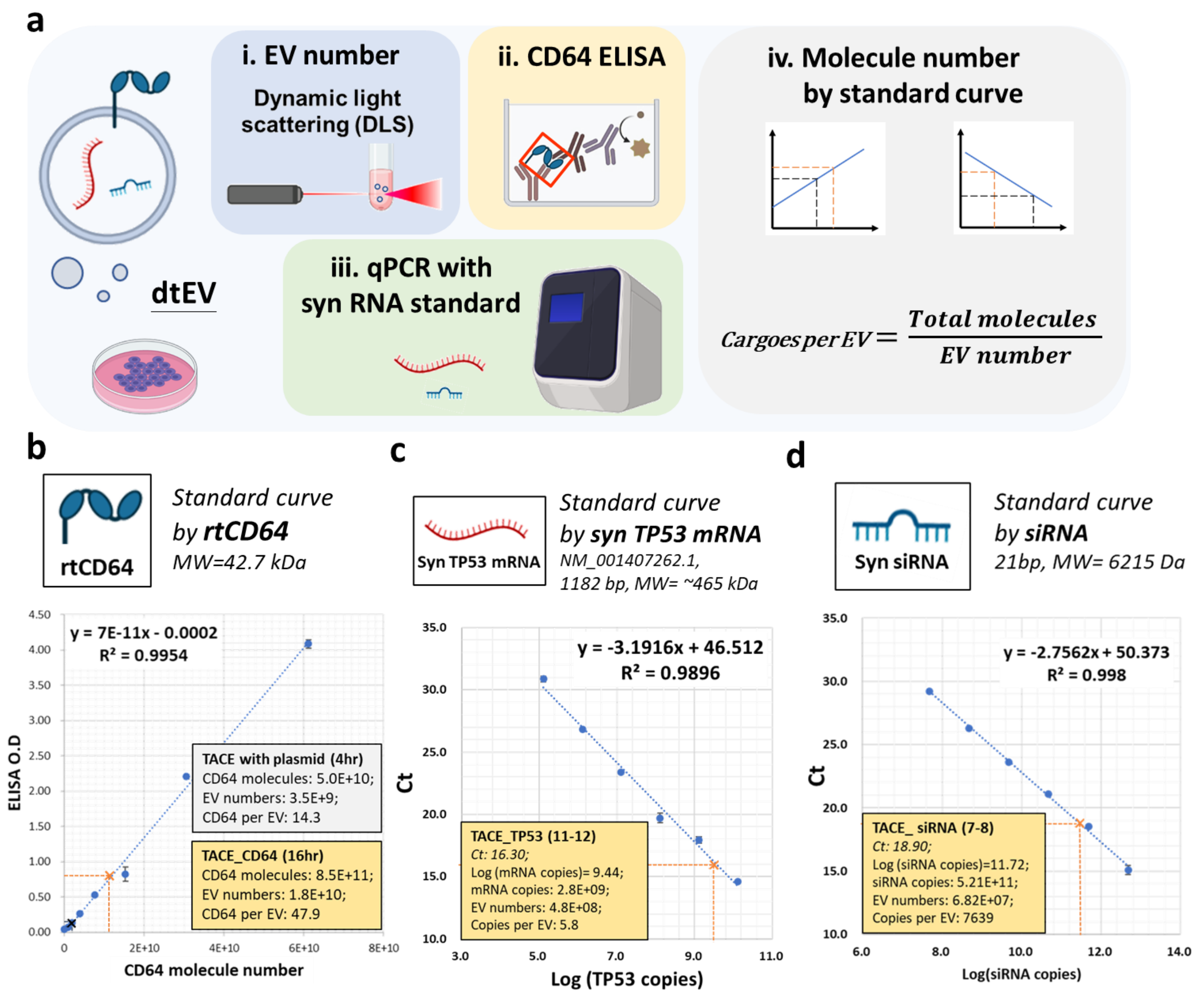

Supplementary Figure 2 | Quantification of cargoes in dtEVs by using calibration curve with standard rtCD64 and synthetic RNAs. a, The dtEVs were harvested from donor cells after TACE treatment. *Step i,* the particle number of dtEVs was first measured by Dynamic light scattering (DLS). *Step ii*, the CD64 molecule was determined by enzyme-linked immunosorbent assay (ELISA). *Step iii*, the copy number of TP53 mRNA and siRNA of KRAS^{G12D} were quantified by Realtime qPCR with calibration curve of synthetic TP53 mRNA (NM_000546.6) and siRNA. *Step iv*, the cargoes per EV was estimated by total amount of each molecule divided by dtEV number. **b,** The standard curve of ELISA was generated by titration of recombinant protein of CD64 (rtCD64. MW = 42.7 kDa) and used to estimated CD64 molecule amount in dtEVs. **c,** The standard curve for TP53 mRNA measurement was generated by synthetic TP53 mRNA (1182 bp, MW ~ 465 kDa). Linearity of calibration was plotted by Ct (cycle threshold) value vs. Log (TP53 copies) and used to estimated TP53 mRNA from dtEV samples. **d,** The standard curve of siRNA was generated by synthetic siRNA of KRAS^{G12D} (21 bp, MW = 6215 Da) and used for the estimated d siRNA of KRAS^{G12D} from dtEV samples. **b-d** Three independent experiments were achieved with the standard curve of ELISA or Realtime qPCR.

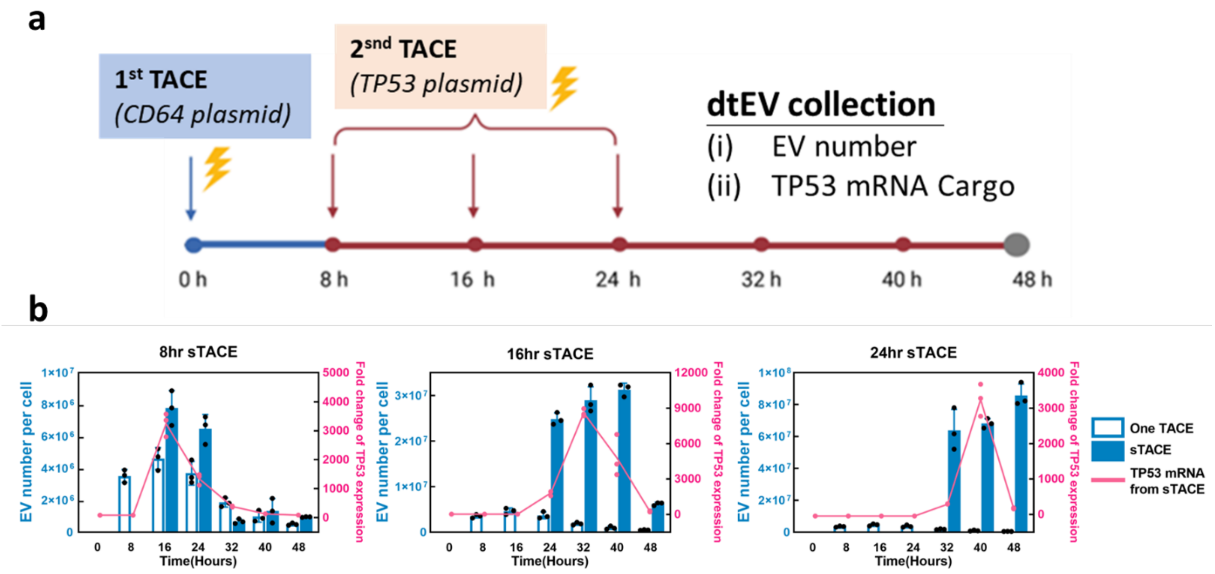

Supplementary Figure 3 | Dynamics of the dtEV production by sequential TACE (sTACE) for donor cell BMSC. a, The sTACE was performed by delivering the CD64 plasmid DNA first and then the TP53 mRNA plasmid DNA with a time-gap of 8 h, 16 h, or 24 h. EV number and TP53 mRNA amount from each sTACE were measured for MEFs or hBMSCs as donor cells. **b,** The sTACE with a 16 h time-gap produced the highest TP53 mRNA cargo loading and EV secretion in the collected dtEVs for donor cell BMSC. All error bars represent SD over three biological independent samples.

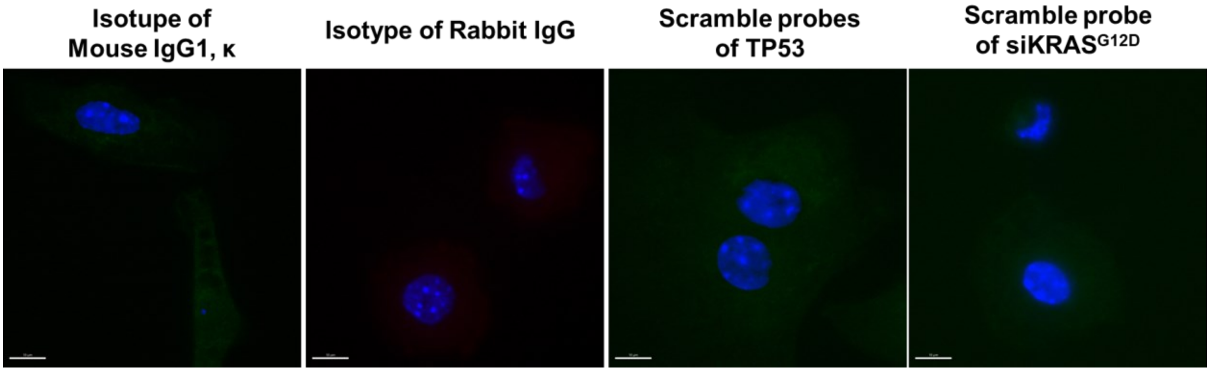

Supplementary Figure 4 | The specificity of the probes and antibodies used for staining of FISH and immunofluorescence in donor cells. The negative control staining of mouse IgG1, rabbit IgG, and scramble probes of TP53 and siKRAS^{G12D} (*scale bar :10 µm*). The sequence of probes for FISH staining are listed in **Supplementary Table 5**. The fluorescent images are representative of three biological independent experiments.

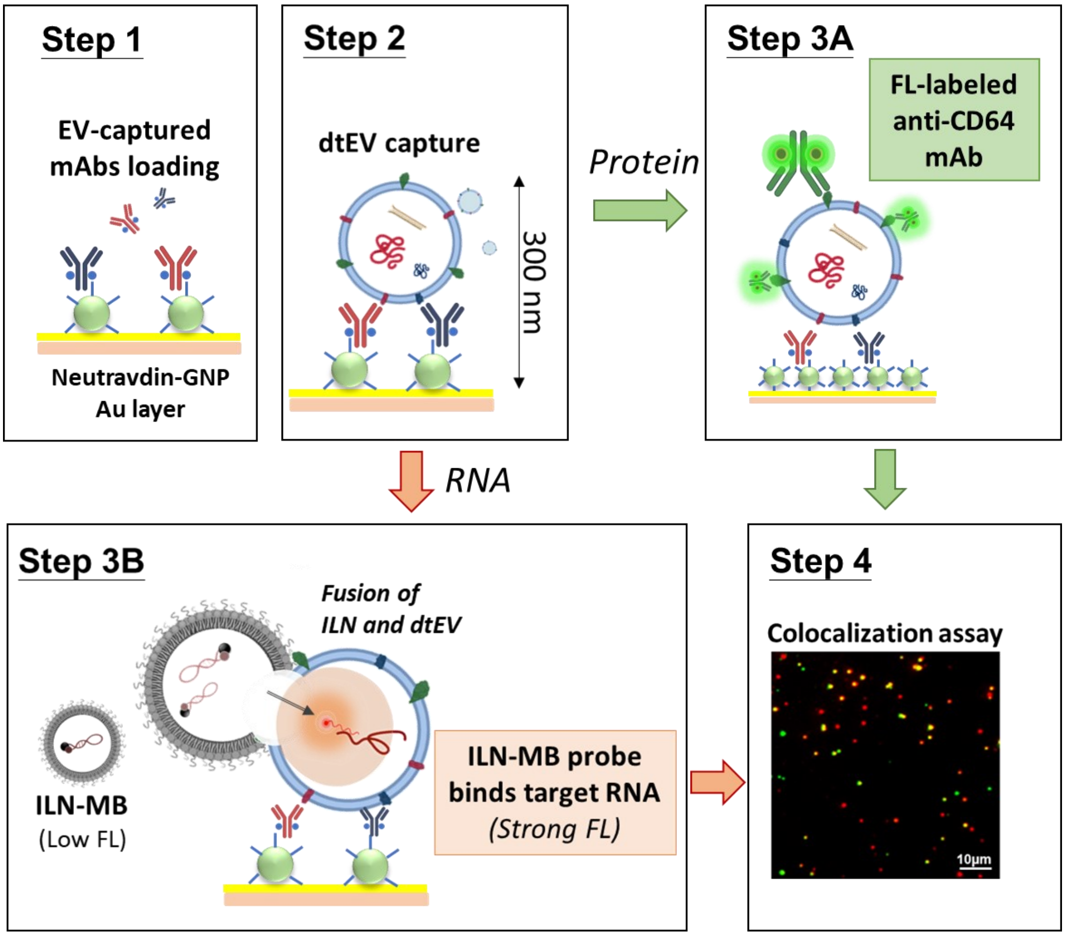

Supplementary Figure 5 | Single dtEV characterization using immune lipoplex nanoparticle (ILN)-Total Internal Reflection Fluorescence Microscopy (TIRFM) assay. (Step 1) Capture antibodies were tethered on a coverglass slide, **(Step B)** which captured selected individual EVs by antibody-antigen affinity. **(Step 3B)** For EV RNA detection, a molecular beacon (MB) for the RNA target was encapsulated in cationic liposomal nanoparticles and added onto the chip after EV capture and washing. The lipoplex-EV fusion led to mixing of RNAs and MBs within the nanoscale confinement near the biochip interface. TIRF microscopy measured the fluorescence signals within 300 nm of the focal plane interface, which is where the captured EVs locate. **(Step 3A)** Similarly, fluorescence labelled detection antibodies were added to the biochip, which would bind the targeted membrane proteins on the captured EV surface. **(Step 4)** By using fluorophores with different colors (wave lengths) on the MBs and detection antibodies, colocalization of RNAs and membrane proteins of interest could be measured. More details of the ILN-TIRF assay can be found in **Supplementary Reference 1** [1].

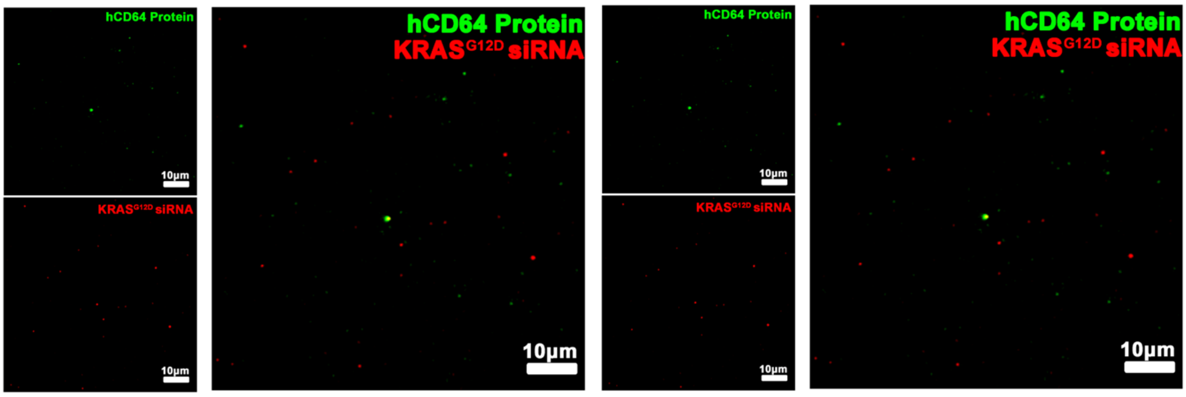

Supplementary Figure 6 | The sequence and specificity of the probes used for ILN assay. The specificity of the probes and antibodies were tested with blank EVs, without human CD64, TP53, or siKRAS^{G12D} expression as negative controls. The design of ILN probes were included in **Supplementary Table 6.** The fluorescent images are representative of three biological independent experiments.

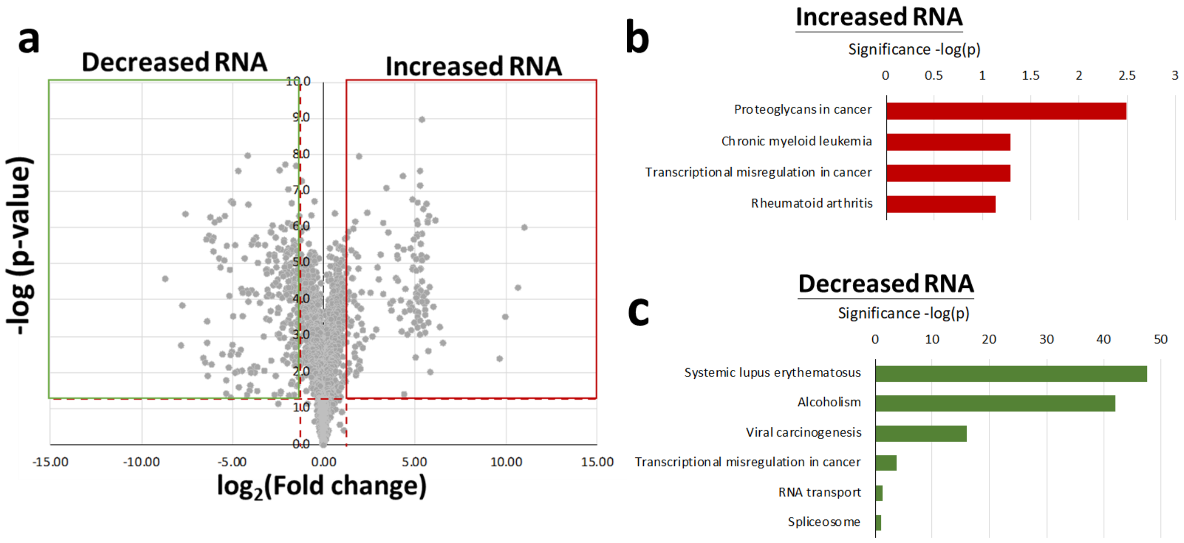

Supplementary Figure 7 | Changes of transcripts in collected EVs from TACE stimulated hBMSCs with TP53 plasmid. a, Changes of transcripts between TACE'ed hBMSCs with or without TP53 plasmid are statically presented in volcano plot by –log(p-value) vs. log2(fold-change). **b,** In comparison to the TACE alone group, the increased transcripts of EVs from cells under TACE with TP53 plasmid were mainly associated with cell cycle related pathways. **c,** In comparison to the TACE alone group, the decreased transcripts of EVs from cell treated with TP53 plasmid were mainly associated with general metabolic processes. The volcano plot was achieved with three biological independent experiments.

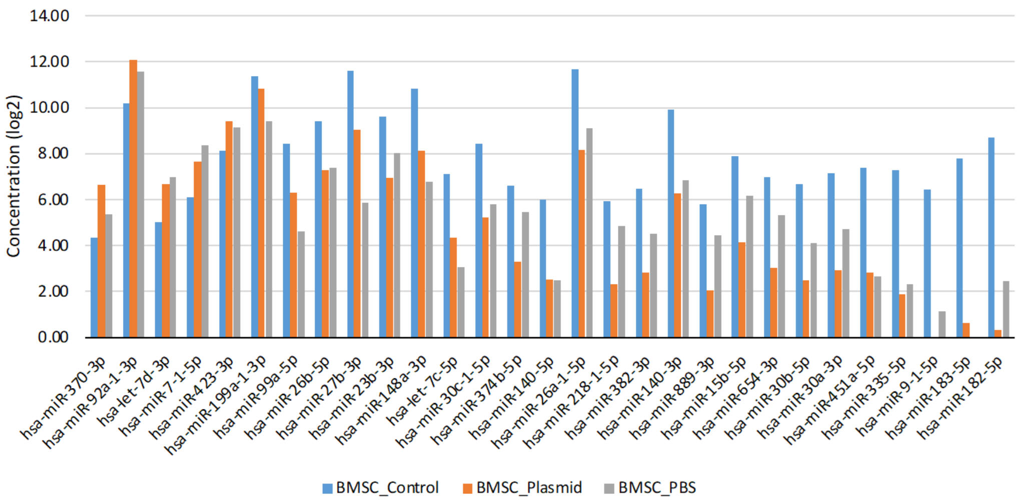

Supplementary Figure 8 | Changes of major microRNAs in EVs from TACE stimulated hBMSCs with PBS or TP53 plasmid. Based on the degree of changes and concentrations in EVs, these miRNAs exhibited the higher expressions before and after TACE. The fold changes (log_2) of major microRNAs are listed in **Supplementary Table 7.** The fold change plot was achieved of three biological independent experiments.

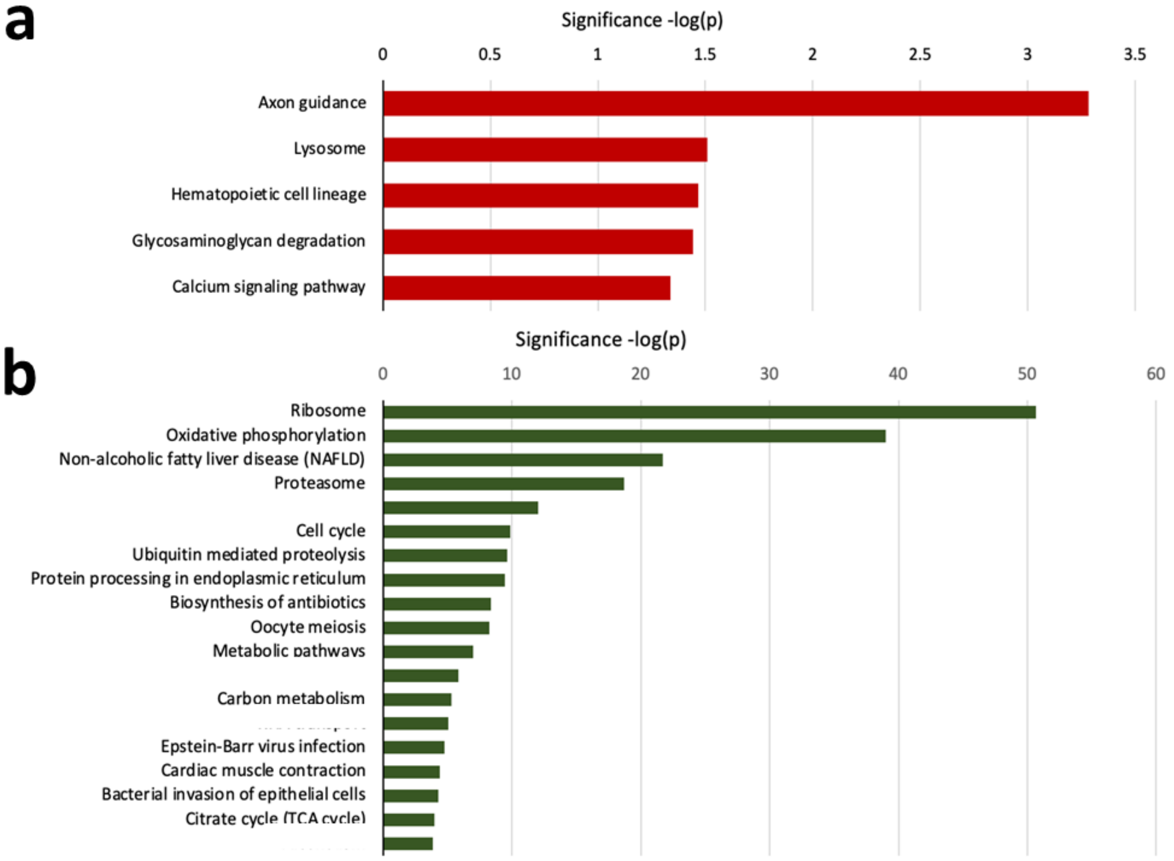

Supplementary Figure 9 | Changes of transcripts in collected EVs from TACE stimulated MEFs with TP53 plasmid. Changes of transcripts between TACE'ed MEFs with or without TP53 plasmid are statically analyzed in volcano plot by –log(p-value) vs. log2(fold-change). Similar affected pathways (highlighted in red and green bars) can be seen between MEFs and hBMSCs (see **Supplementary Figure 6**) **a,** Comparing to TACE alone group, the increased transcripts of EVs from cells under TACE with TP53 plasmid were mainly associated with cell cycle related pathways. **b,** In comparison to TACE alone group, the decreased transcripts of EVs from cell treated with TP53 plasmid were mainly associated with general metabolic processes. The fold change plot was achieved by three biological independent experiments.

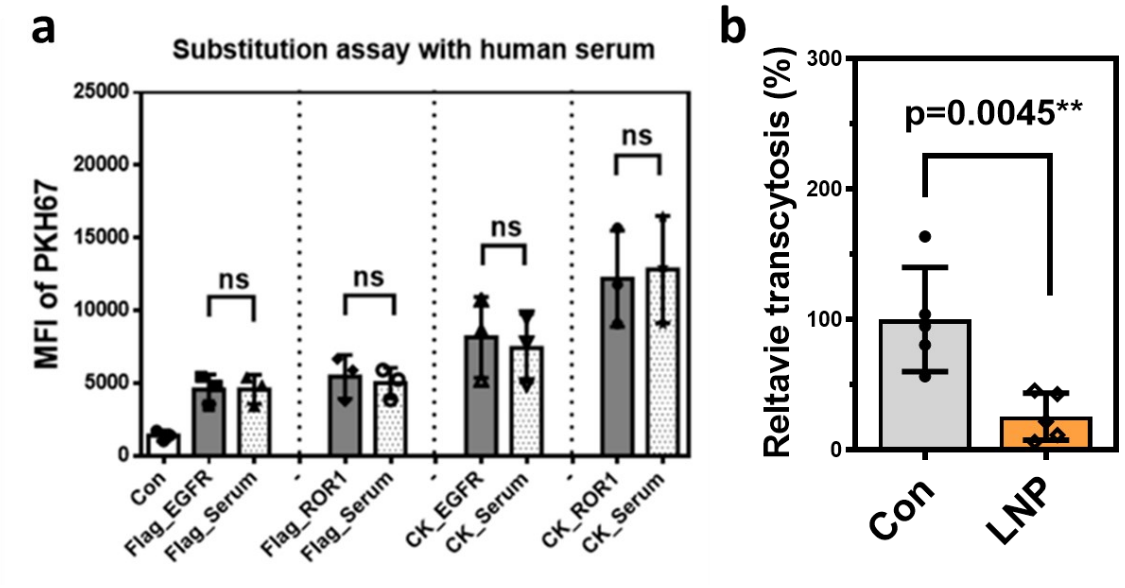

Supplementary Figure 10 | Stability of hmAbs on EVs and penetration comparison of different nanocarriers in PANC-1 spheroids. a, A substitution assay demonstrates the stability of preloaded humanized antibodies (αROR1 and αEGFR) on EVs in 50% human serum. (n= 3 biological independent experiments) **b,** Bottom cells took up over 2-fold more dtEVs (Con) than LNPs from top cells in the Transwell® transcytosis assay. (n = 5 biological independent experiments). Data is presented as mean ± SD.

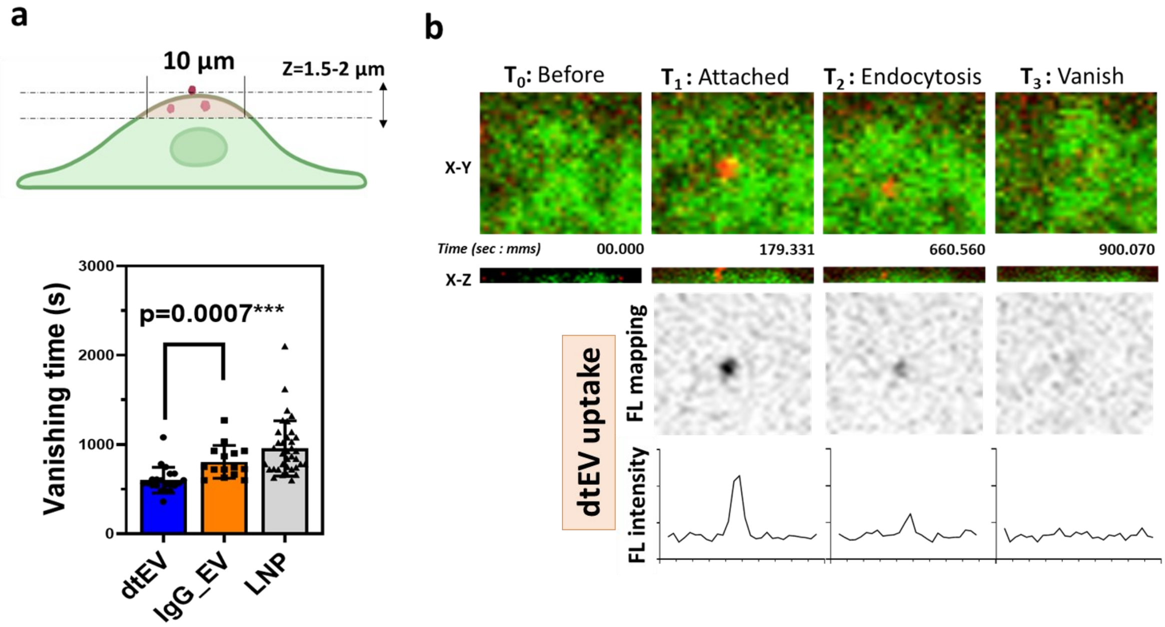

Supplementary Figure 11 | Dynamics of single-EV analysis reveals dtEVs could accelerate endocytosis in PANC-1 cells. Live cell confocal imaging was conducted by an inverted motorized microscope (Nikon TiE, Japan) coupled to an environmental enclosure (OkoLab, Italy). The temperature was set at 36°C throughout the entire time of experiment. Confocal images were acquired by an EMCCD camera (Andor iXON885, UK) through a 60X or 100X objective (Nikon VC, NA = 1.4) and spinning-disk head (CSU-X1, Yokogawa, Japan). Illumination of lasers was controlled by an iLAS2 (GATACA Systems, France) and Metamorph (Molecular Devices, USA). Nanoparticles (NPs) including dual- and single-targeted extracellular vesicles (EVs) and non-targeted lipid nanoparticles (LNPs) were labelled by either PKH67® or CellVue® Claret Far Red Fluorescent Cell Linker kit (Merck, USA) according to manufacturer's indications. PANC-1 cells were plated on round cover-glass (18 mm, Marienfeld, Germany) and cultured for 48-72 h before the plasma membrane stained by Cell Mask® Green followed by addition of various NPs. A series of z-axis confocal images (4-6 µm, distance of each step = 0.5 µm) were taken focusing at Cell Mask-stained apical cones illuminated by laser 491 nm. NPs, including dtEVs), IgG-EV, and LNPs labelled by CellVue® were added to the cell medium and subsequently excited by laser 639 nm for multi-positional image acquisition. **a**, Multi-parametric confocal images were alternatively recorded at each channel every minute for 60 min. Confocal image sections projected to one Z plane with clear apical membrane features and attaching nanoparticles were chosen for monitoring NP's successive trajectory paths and calculating the vanishing time, which was measured by subtracting the time of disappearance of a single nanoparticle to that of its presence in the films. **b,** The vanishing time was calculated between T_1 to T^3 (720.7 second). An intensity map of NP

was established to confirm the endocytosis event. **c,** The shorter vanishing time of dtEV up-taken by PANC-1 suggested an accelerated endocytosis. [dtEV: 600.2±31.46; IgG_EV: 805.6±47.59; LNP: 956.2±51.47]. Data is presented as mean ± SD from three independent experiments**.**

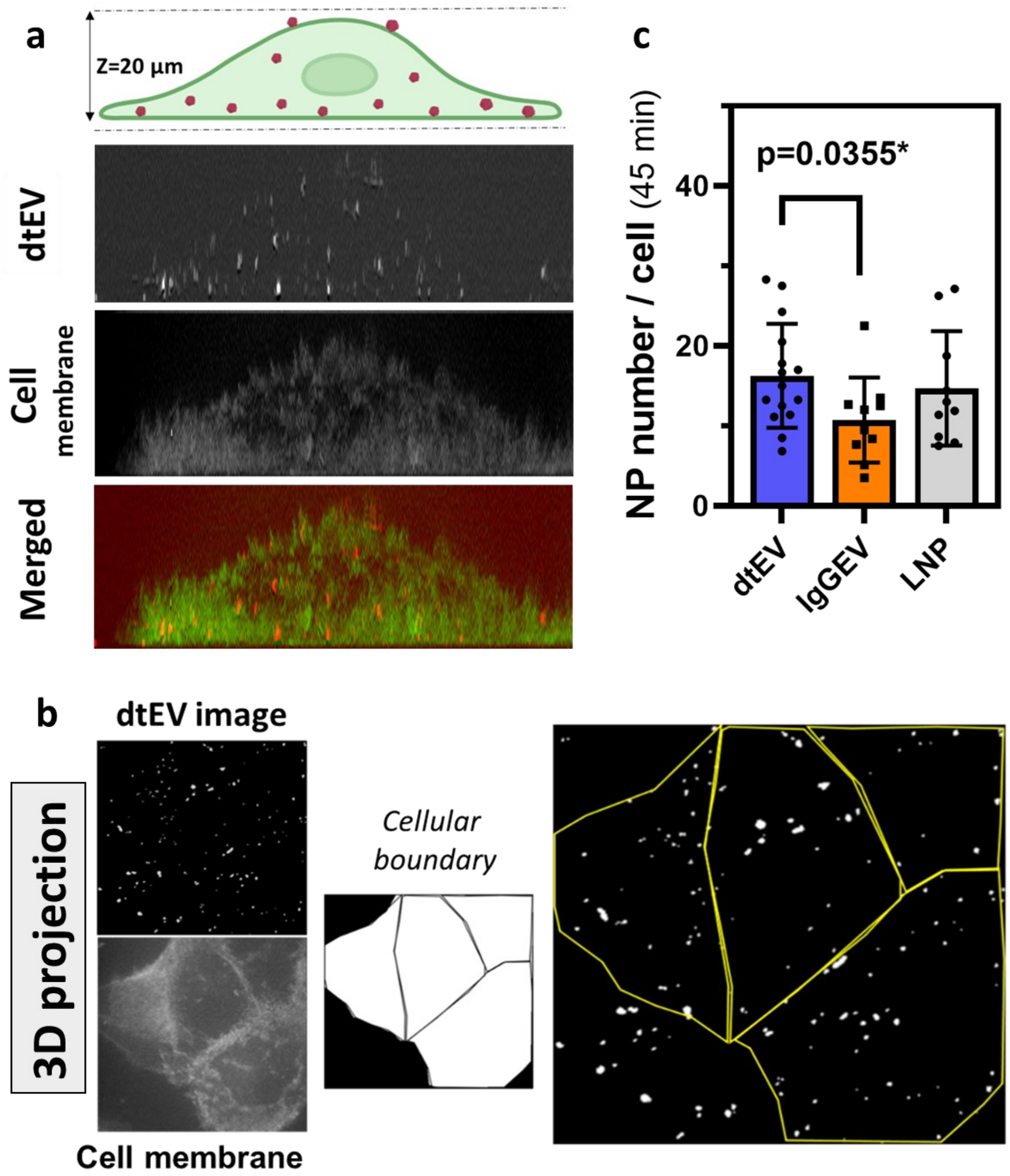

Supplementary Figure 12 | Three-dimensional (3D) quantification of single cells reveals enhanced dtEV accumulation in living PANC-1 cells. a, An end-point test was operated by allowing the uptake of NPs with living cells for 45 min and acquiring the dual-color confocal images for the entire cell volume (Z-axis = 20 µm). **b,** The z-stack images were projected to one Z plane, individual cell contour was subdivided by cell mask signals, dots inside the cells were binarized and their numbers were counted by Metamorph and Image J. **c**, For nanoparticles uptake within 45 min, PANC1 cells are capable of engulfing and storing dtEVs (18.42±9.48) more efficiently than IgG_EV (13.03±8.67) or LNP (15.12±12.24). Data is presented as mean ± SD from three independent experiments**.**

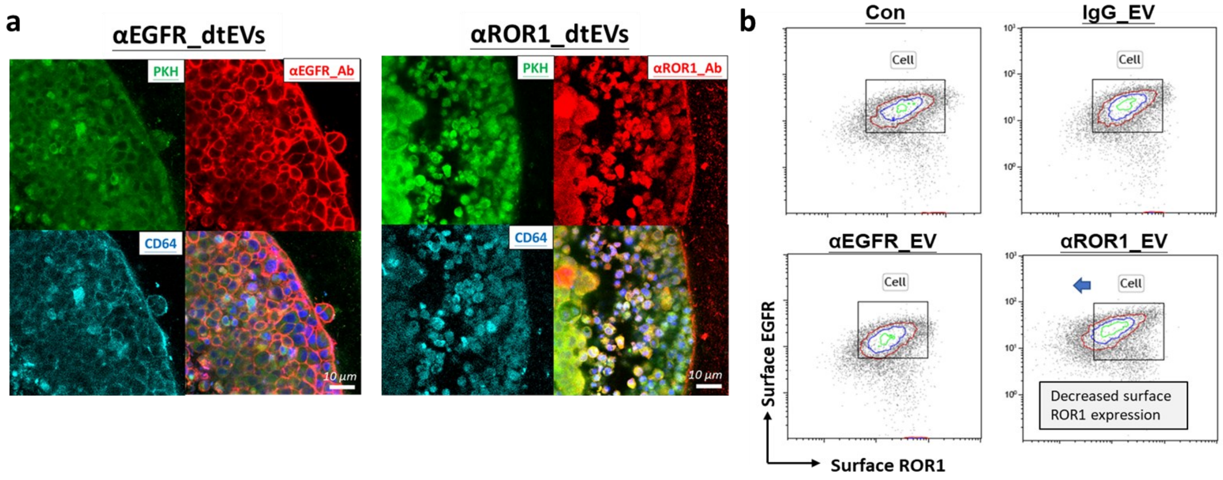

Supplementary Figure 13 | Targeting and internalization ability of dtEVs with αEGF or αROR1 in PANC-1 spheroid. a, The PANC-1 tumor spheroids were treated with PKH67 labeled dtEVs formulated with αEGFR or αROR1 for 4 hr. The distribution of dtEVs in tumor spheroids was monitored by three compartments as PKH67-labeling (green), loaded hIgG (red, anti-hIgG for αEGFR/αROR1), and anti-CD64 antibodies (cyan). Formulation of αROR1_dtEV showed stronger internalization with evidenced by cytosolic dtEV signals. **b,** The PANC-1 cells from treated spheroids were measured for their surface expression of EGFR and ROR1, respectively. Decreased ROR1 expression, but not much of EGFR, was found on the surface of PANC-1 cells, suggesting a stronger internalization ability of dtEVs with αROR1 formulation. Representative fluorescent images and flow data are shown from three independent experiments.

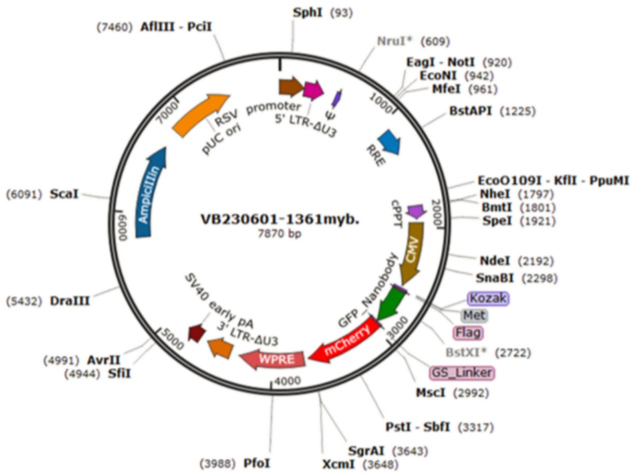

Anti-GFP_mCherry

MDYKDDDDKVQLVESGGALVQPGGSLRLSCAASGFPV

NRYSMRWYRQAPGKEREWVAGMSSAGDRSSYEDSV

KGRFTISRDDARNTVYLQMNSLKPEDTAVYYCNVNVG

FEYWGQGTQVTVSSGSGSGSGSVSKGEEDNMAIIKEFMR

FKVHMEGSVNGHEFEIEGEGEGRPYEGTQTAKLKVTK

GGPLPFAWDILSPQFMYGSKAYVKHPADIPDYLKLSFPE

GFKWERVMNFEDGGVVTVTQDSSLQDGEFIYKVKLR

GTNFPSDGPVMQKKTMGWEASSERMYPEDGALKGE

IKQRLKLKDGGHYDAEVKTTYKAKKPVQLPGAYNVNIK

LDITSHNEDYTIVEQYERAEGRHSTGGMDELYK

Flag Linker

αGFP_Nanobody mCherry

Supplementary Figure 14 | The design of the anti-GFP_mCherry fluobody. The fluobody includes an anti-GFP nanobody link with mCherry fluorescent protein as indicator and would be expressed by CMV promotor.

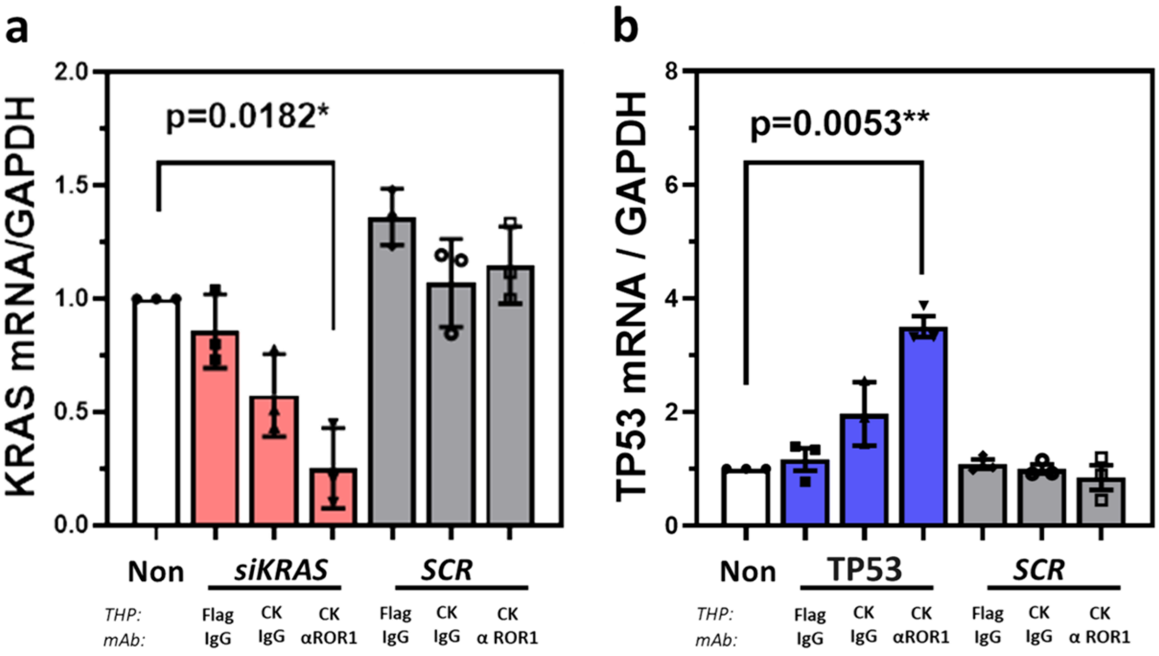

Supplementary Figure 15 | *In vitro* therapeutic efficacy results of various EVs in PANC-1 cells. a, Real-time qPCR assay of PANC-1 cells treated with various dtEVs carrying siKRAS^{G12D} or scramble siRNA (SCR) for 24 h showed silenced KRAS mRNA expression (n = 3 biological independent samples). **b,** Real-time qPCR assay of PANC-1 cells treated with various dtEVs carrying either TP53 or scramble (SCR) mRNA for 24 h. The p53 expression was upregulated in cells treated with dtEVs carrying TP53 mRNA (n = 3 biological independent samples).

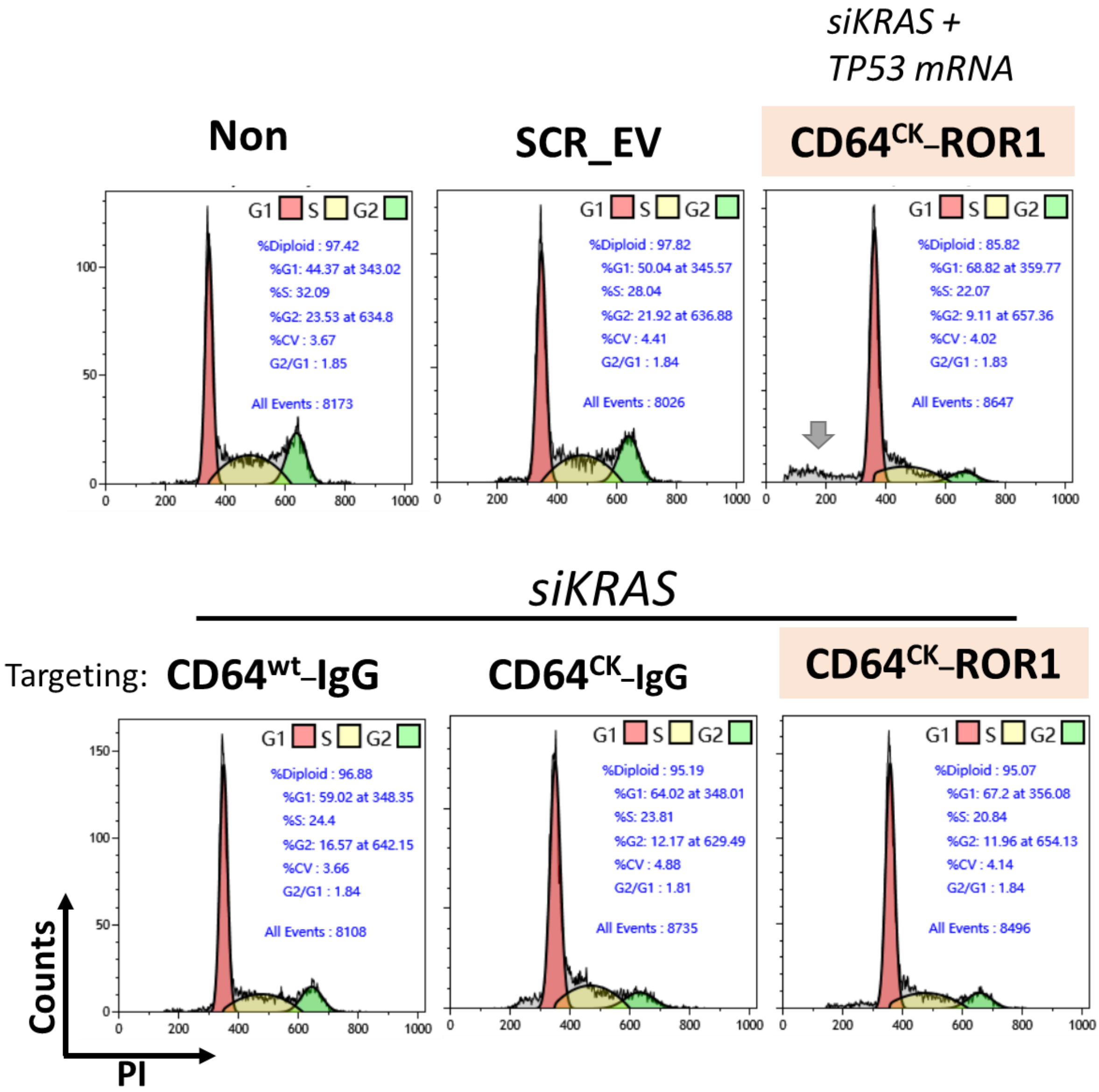

Supplementary Figure 16 | Cell cycle analysis by PI staining in PANC-1 cells with dtEV treatment. The cell cycle analysis was analyzed by intensity of PI staining for G1 (red), S (yellow) and G2 (green) stages. The treatment of dtEVs encapsulating siKRAS^{G12D} and TP53 mRNA increased cell cycle arrest (G1, red) and induced apoptosis (fragmental DNA, gray) in PANC-1 cells. Lower panel shows cell cycle regulation in PANC-1 cells treated with various formulation of dtEVs (CD64^{WT}, CD64^{CK}, IgG, or αROR1) carrying siKRAS^{G12D} alone. Dual-targeted delivery of siKRAS^{G12D} through dtEVs (CD64^{CK} + αROR1) enhanced siKRAS^{G12D}-induced cell cycle arrest effect. The flow data is representative of four independent experiments.

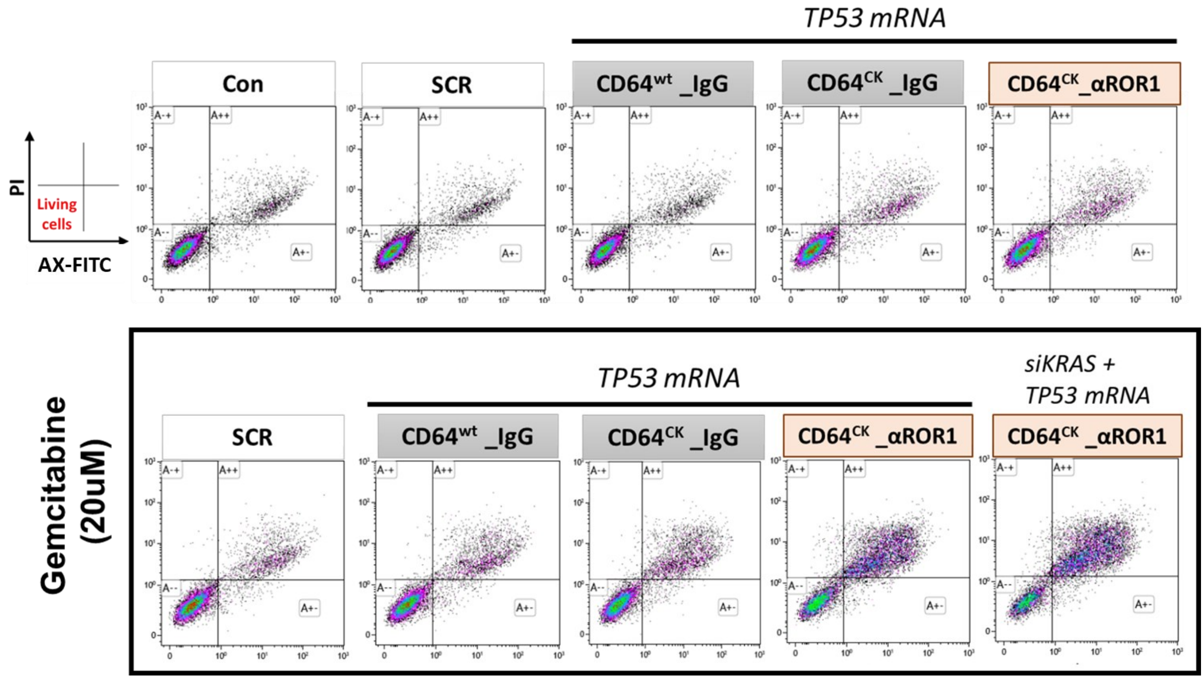

Supplementary Figure 17 | Therapeutic effects of dtEVs on viability of PANC-1 cells. PANC-1 cells were treated with various formulations of EVs ($CD64^{WT}$, $CD64^{CK}$, IgG, or αROR1) or dtEVs carrying TP53 mRNA for 48 h. Live, dead, and apoptotic populations were determined by Annexin V/ PI staining. Low dose of Gemcitabine (GEM, 20 μM) was applied as combinative therapy with dtEVs (black box). Combination of GEM and dtEVs with $siKRAS^{G12D}$ and TP53 mRNA led to high cell death in PANC-1 cells. Con is EVs from untreated native MEFs. The flow data is representative of four independent experiments.

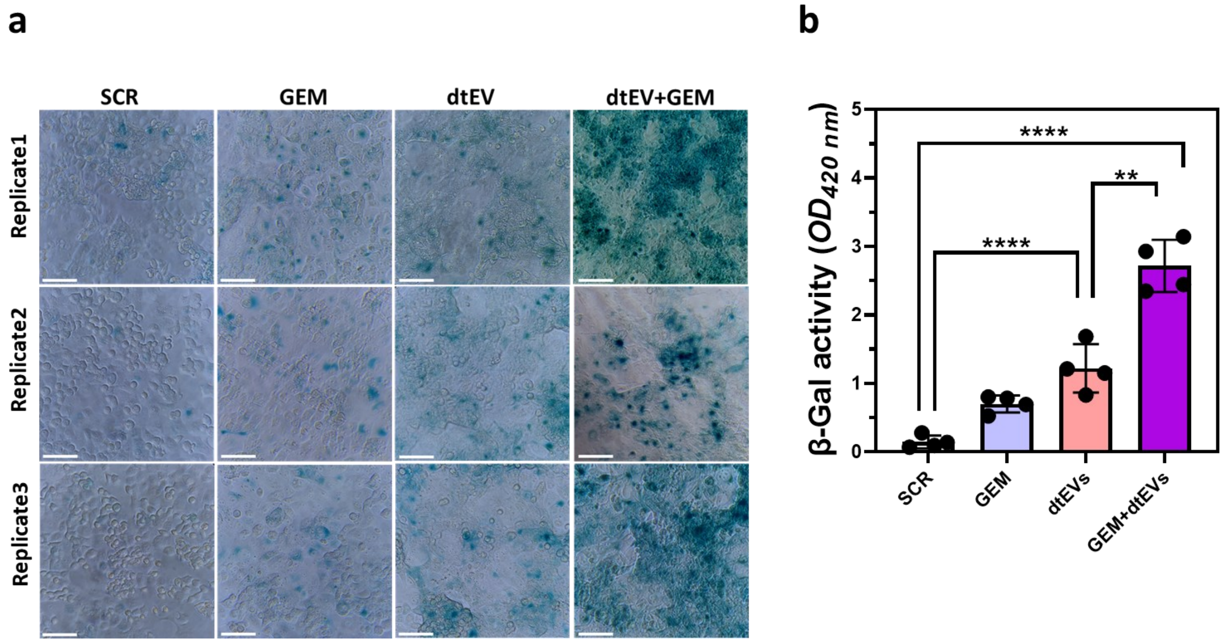

Supplementary Figure 18 | The SA-β-galactosidase (SA-β-gal) activity assay showed dtEV-induced OIS. a, We conducted the SA-β-gal activity assay on cultured PANC-1 cells to investigate OIS. **b**, The quantification of β-galactosidase activity was measured at OD420nm. Following a 24-h treatment, we observed a robust induction of OIS by dtEVs, both with and without Gemcitabine treatment, as evidenced by approximately a 10-fold increase in SA-β-gal activity compared to the control groups [Scramble: 0.14 ± 0.09; GEM: 0.70 ± 0.125; dtEV: 1.22 ± 0.35; dtEV+GEM: 2.714 ± 38.12]. Data were presented as mean ± SD from four independent samples**.** (scale bar: 100 μm)

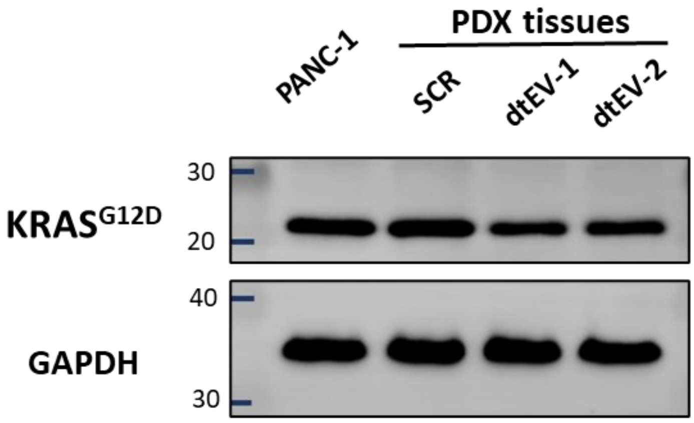

Supplementary Figure 19 | dtEVs suppressed KRAS expression in tumours from orthotopic PANC-1 mouse model. We performed Western blotting on the archived tumour tissues obtained from orthotopic PANC-1 mouse model. The knockdown of KRAS expression was validated by using anti-KRAS^{G12D} (clone: HL10) antibodies, with a comparison to the scramble control group (SCR). The PANC-1 cell line was utilized as a positive control. The western blots are representative of the PDX tumour tissues from three mice of each treatment group.

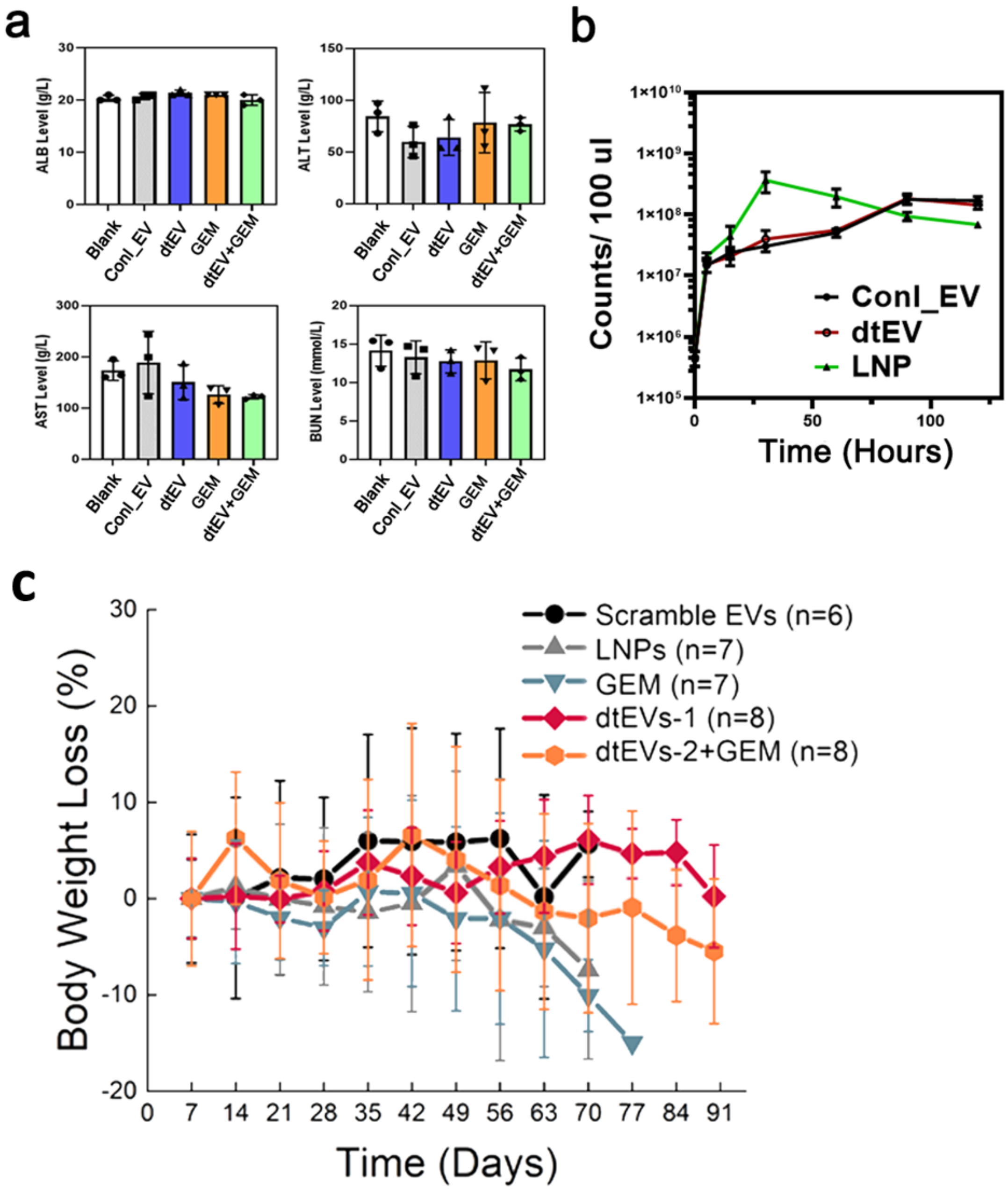

Supplementary Figure 20 | Toxicity and blood circulation of dtEVs *in vivo*. a, Immunogenicity in treated mice shows little liver toxicity of dtEVs with low-dose GEM. **b,** Nanocarrier concentration changes in blood circulation after IP injection. Conl_EV: non-targeted EVs**.** All error bars represent s.e.m. over three independent samples. **c,** animal body weight loss. **a, b** Data is presented as mean ± SD from three mice in each experiment**.**

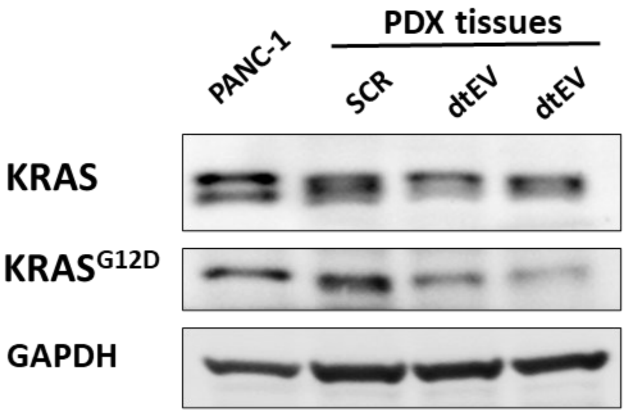

Supplementary Figure 21 | dtEVs suppressed KRAS expression in PDAC tumours from PDX mice. We performed Western blotting on the archived tumour tissues obtained from the PDX animal studies. The knockdown of KRAS expression was validated using anti-KRAS (clone: AT2F8) and anti-KRAS^{G12D} (clone: HL10) antibodies, with a comparison to the scramble control group (SCR). The PANC-1 cell line was utilized as a positive control. The western blots are representative of the PDX tumour tissues from three mice of each treatment group.

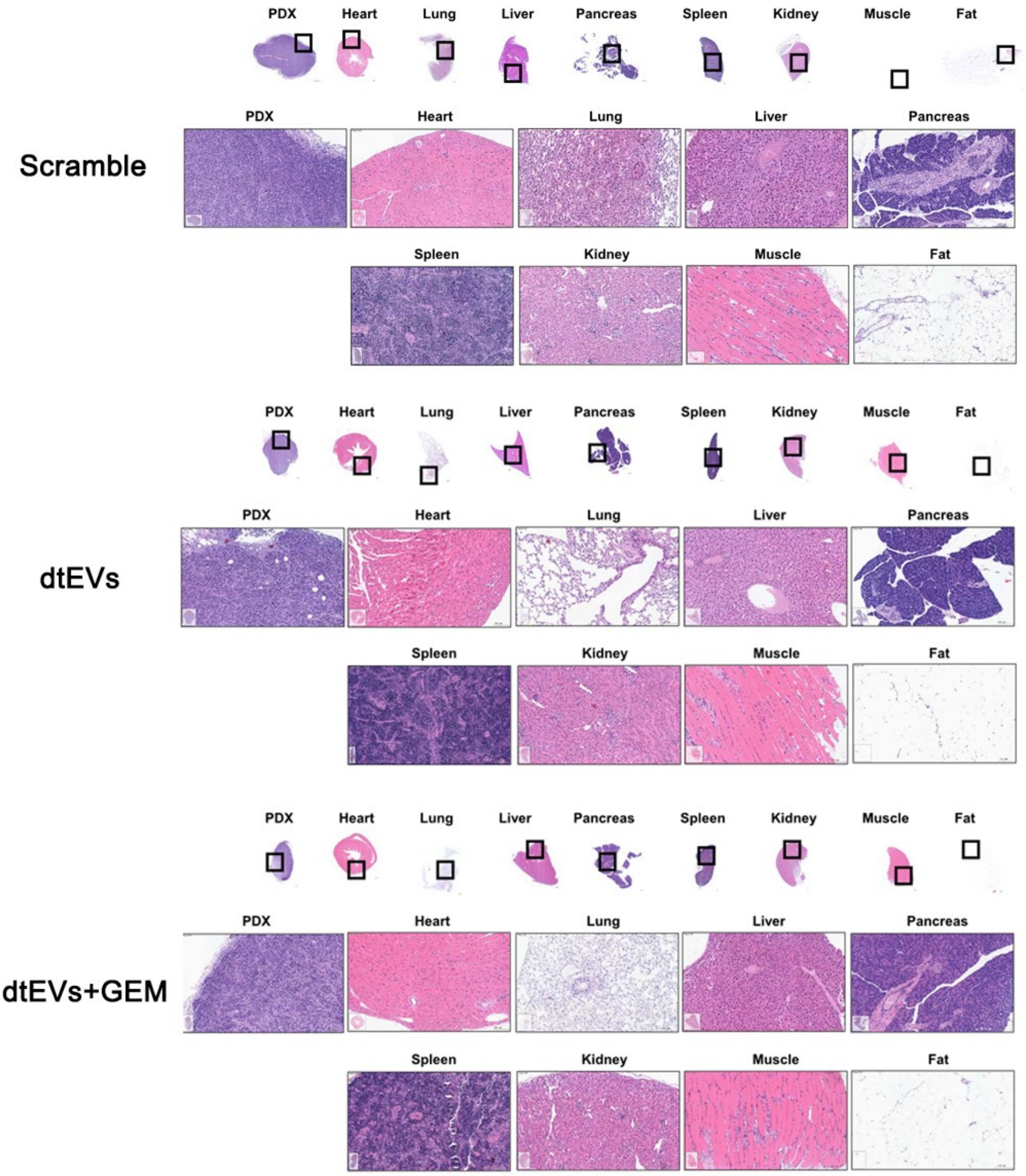

Supplementary Figure 22 | Histological analysis by hematoxylin and eosin (H&E) staining of tumour and main organs from subcutaneous PDX mice. In addition to the PDX tumour, cancer lesions were found in the lung, pancreas, and fat tissue of sacrificed mice after treatment. Applying dtEVs, particularly in combination with Gemcitabine (GEM), could substantially reduce the lesion number and size in those organs. The histological analysis was representative of orthotopic tumour tissues from 3 mice of each treatment group.

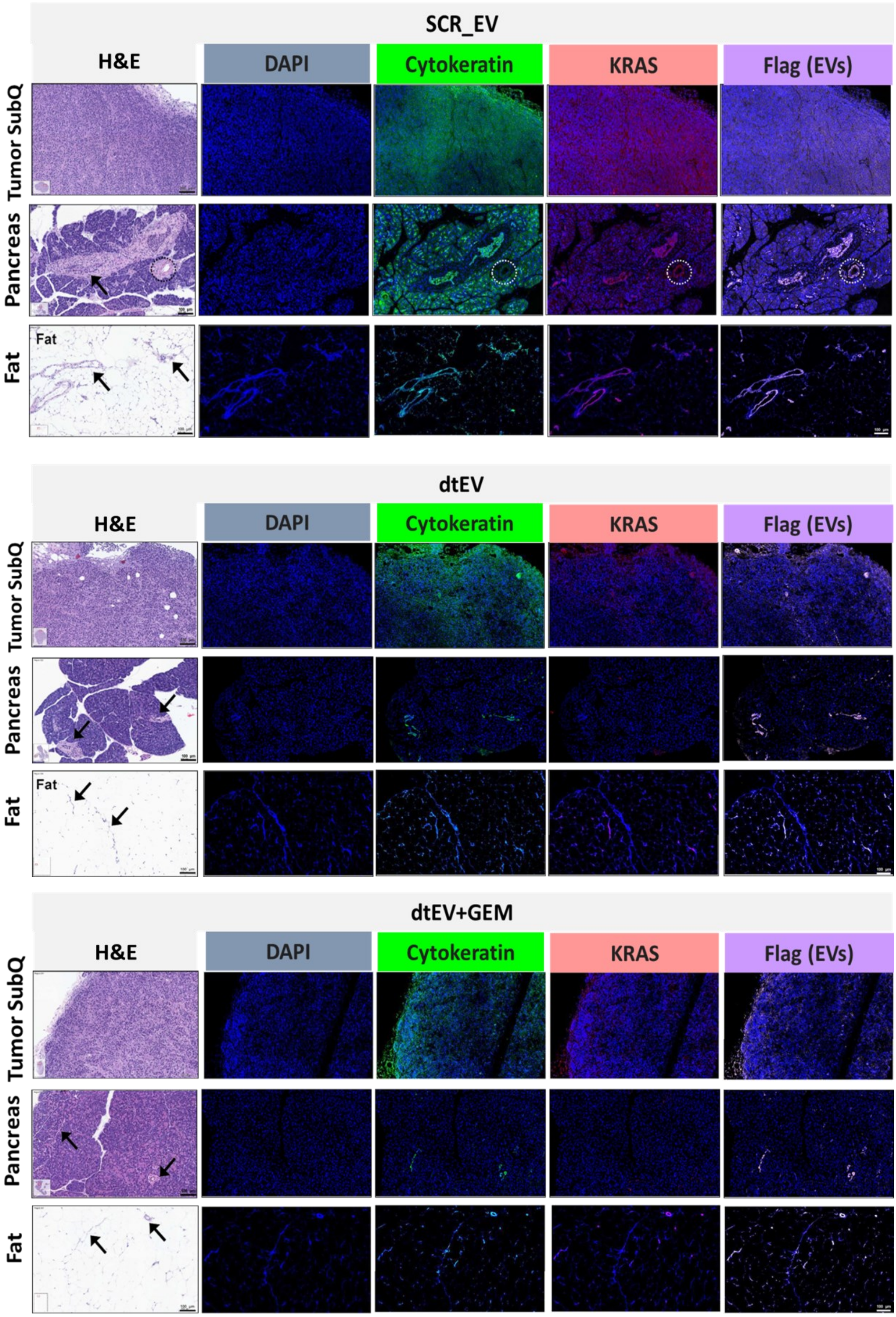
SCR_EV
dtEV
dtEV+GEM
H&E
DAPI
Cytokeratin
KRAS
Flag (EVs)
Tumor SubQ
Pancreas
Fat

Supplementary Figure 23 | Pathological analysis for the tissue sections from dtEV-treated subcutaneous (subQ) PDX mice. Tissue sections from subQ tumour, metastatic pancreas, and fat tissue from treated PDX mice were stained with hematoxylin and eosin *(*H&E) stain, DAPI (blue, nuclei), anti- cytokeratin (green, tumour marker), anti-KRAS (red), and anti-Flag (purple, delivered EV marker). According to H&E and cytokeratin (green) staining, both original subcutaneous tumour and metastatic lesions in the pancreas and fat tissues were decreased by dtEVs with or without GEM. By staining of Flag (purple), we observed dtEVs could accumulate in both original subcutaneous tumour and metastatic lesions of the pancreas and fat tissues. The histological analysis was representative of orthotopic tumour tissues from 3 mice of each treatment group. (*scale bar: 100 µm*)

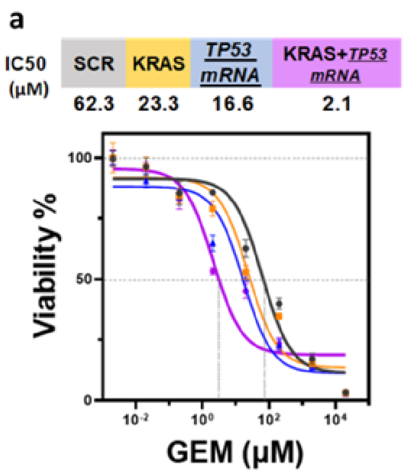

b

Target gene	Target sequence RNAi
TM4SF1	CTGTGCATCGCGGCTAATATTTATT
GLI1	GCTCAGCTTGTGTGTAATTAT
MUC1	GACACAGTTCAATCAGTATAA
STK33	GTCGTAATGTACATGTTATT
MGMT	GAGCAGGGTCTGCACGAAATA

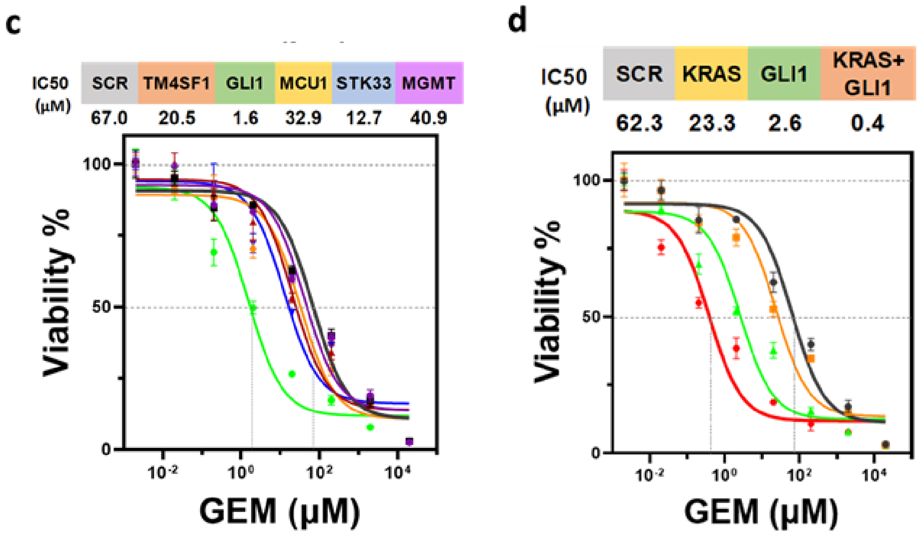

Supplementary Figure 24 | Analysis of combinative gene therapy against intrinsic chemoresistance of PANC-1 cells to Gemcitabine (GEM). Gemcitabine-resistant PANC-1 cell line was treated with dtEVs ($5x10^6$ EVs/cell) carrying various siRNAs and/or TP53 mRNA. Cell viability was determined by MTT assay after doses titration of GEM for 48 h, and then IC50 was calculated. **a,** Combinative dtEVs (*purple curve*) carrying siKRAS^{G12D} and wildtype TP53 mRNA sensitized the PANC-1 cells to Gemcitabine. **b,** Referring to the database of Cancer Therapeutics Response Portal, we select five potential targets associated with gemcitabine-resistance in PDAC patients. The target sequences of each siRNA design are listed. **c,** Among the five siRNAs, suppressing the GLI1 expression (*green curve*) strongly decreased the Gemcitabine-resistance in PANC-1 cells. **d,** Co-suppressing KRAS^{G12D} and GLI1 pathways by combined siRNAs (*red curve*) greatly decreased the Gemcitabine-resistance in PANC-1 cells. All error bars represent s.e.m. over three independent samples. The siRNA sequences are given in **Supplementary Table 3**.

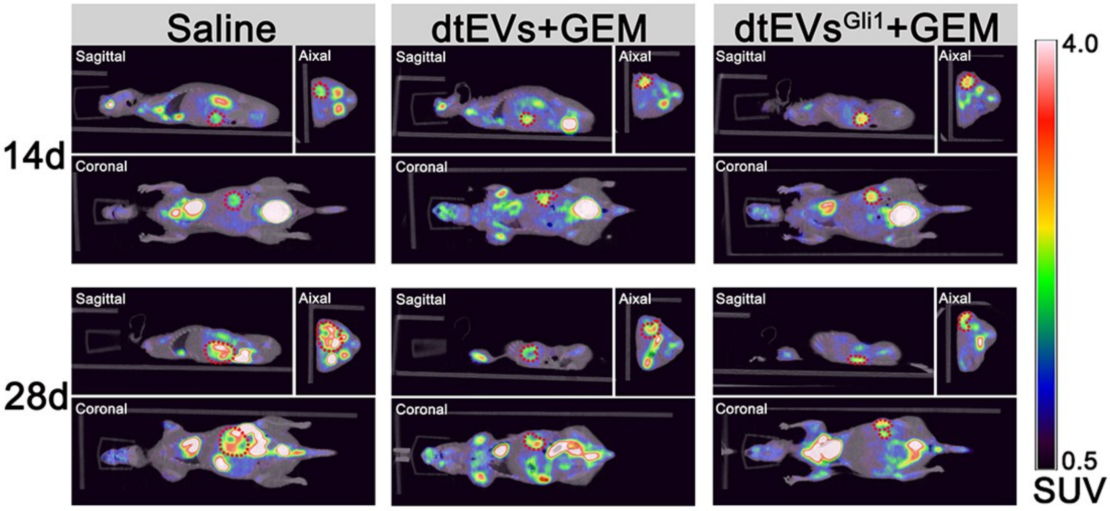

Supplementary Figure 25 | Tumour size measurements by positron emission tomography and computed tomography (PET-CT) in orthotopic PDX PDAC mice. The orthotopic tumours were positioned through three corners including sagittal plane, axial plane, and coronal plane. (Red dot lines: tumour site). Representative PET-CT images displaying orthotopic PDAC tumors in the abdominal cavity of mice (n=4 mice each group).

a

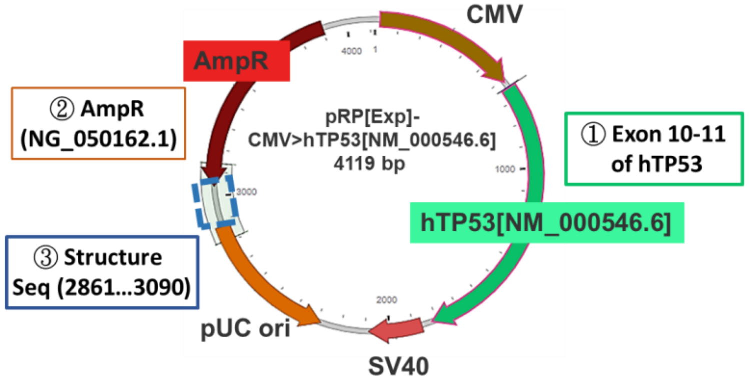

b

Primers for Structural DNA

Forward:
5'-*TTTTCTACGGGGTCTGACGC*

Reverse:
5'-*CGCTGAGATAGGTGCCTCAC*

c

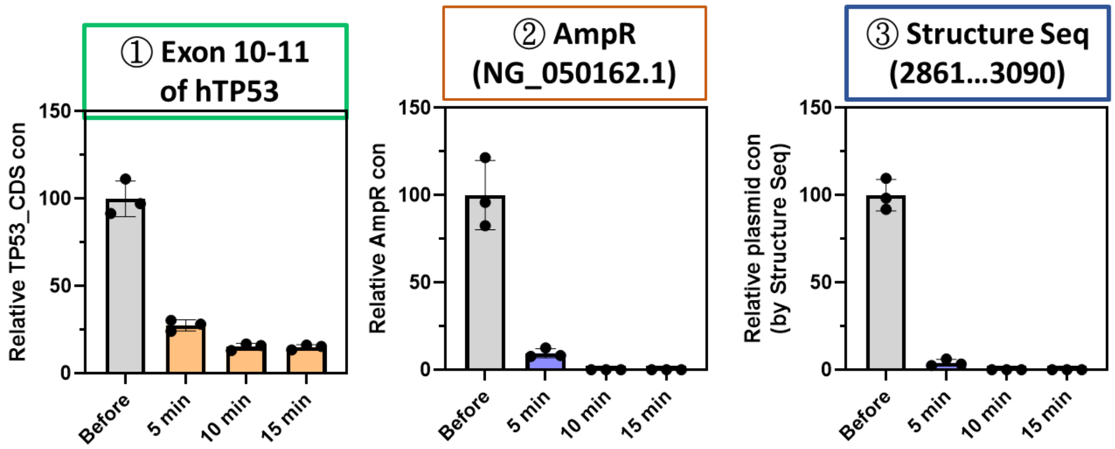

Supplementary Figure 26 | The DNase treatment is essential for removal of the plasmid DNA contamination in dtEV samples. a, The plasmid DNA was measured by three different qPCR primer sets for (i) Exon 10-11 of hTP53 (*green*, Hs01034249_m1, ThermoFisher), (ii) AmpR (*red*, Mr00661613_cn, Thermofisher), and (iii) structural sequence of plasmid DNA (blue). The qPCR measurement of Exon 10-11 of hTP53 can detect both plasmid DNA and mRNA of TP53, while the detection region of AmpR and Structure Seq only exists in plasmid DNA. b, The primer set was designed to detect the region of 2861 to 3090 bp from full length plasmid DNA (4119 bp) by qPCR assay. **c,** Effect of the DNase treatment time (5, 10, or 15 min) on dtEV purification determined by EV RNA extraction for qPCR assay. A 15 min DNase treatment time could remove the plasmid DNA well without affecting the mRNA content in dtEVs. Less than 0.001% of plasmid DNA, either AmpR or structure sequence of plasmid, were detectable after the 15 min DNase treatment. Data is presented as mean ± SD over three biological independent experiments.

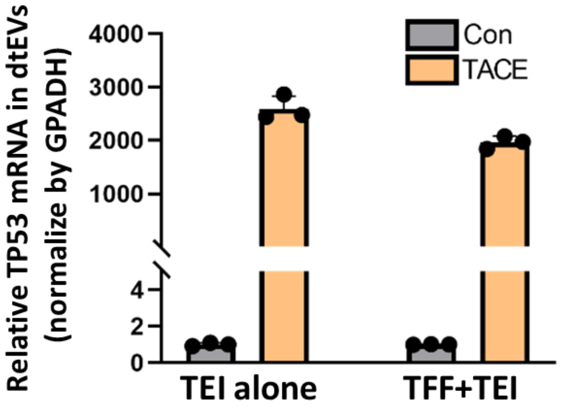

Supplementary Figure 27 | Comparison of RNA loading in the dtEVs collected from TEI isolation reagent and TFF system. we conducted a side-by-side comparison of TFF and TEI methods. After TACE, the culture medium was initially cleared of cell debris and used for TFF comparison. Half of the purified EV solution was utilized to estimate the EV yield with TFF, while the other half was further concentrated using the TEI reagent kit. We observed that the loading efficiency for RNA was similar when using either TFF or TEI for qPCR analysis. Data is presented as mean ± SD over three biological independent experiments.

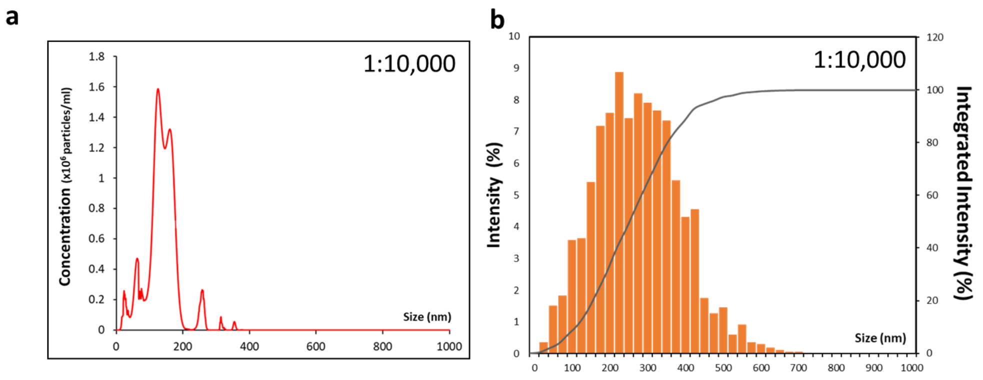

c

Dilution	NTA	DLS
1:10	N.A.	$2.83 \pm 0.46 \times 10^{11}$
1:100	N.A.	$4.69 \pm 0.23 \times 10^{10}$
1:1,000	$1.06 \pm 0.06 \times 10^{9}$	$3.89 \pm 0.21 \times 10^{9}$
1:10,000	$2.78 \pm 0.07 \times 10^{8}$	$5.62 \pm 0.13 \times 10^{8}$
**Estimated concentration**	~1.92×10^{12}	~4.25×10^{12}

Supplementary Figure 28 | Comparison of dtEV measurement between DLS and NTA. Each method has its own limitations in terms of detection. NTA requires serial dilution to match the chamber capacity, typically aiming for 10-100 nanoparticles per frame to track Brownian motion. On the other hand, DLS is suitable for nanoparticle concentrations spanning 4-5 orders of magnitude. In our study, we compared NTA **a,** and DLS **b,** measurements of the dtEVs we collected under the same dilution factor (1:10,000), as depicted in the provided figure. **c,** The concentration measurements obtained by these two methods were comparable, falling within a similar order of magnitude. However, it is worth noting that the serial dilution used in NTA may potentially result in the loss of signals from larger particles (> 500 nm). Additionally, DLS exhibits poor resolution and sensitivity when it comes to particles smaller than 50 nm. Data is presented as mean ± SD over three biological independent experiments.

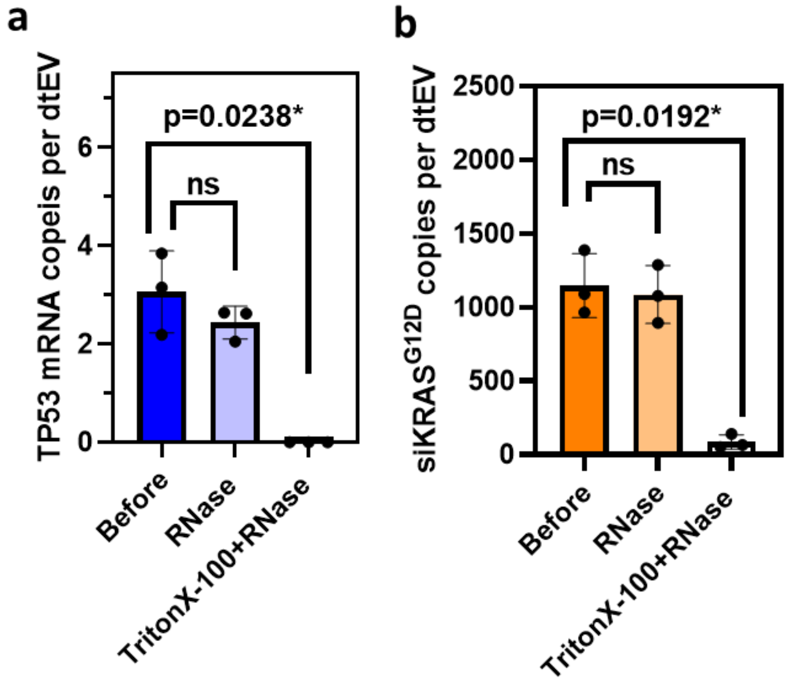

Supplementary Figure 29 | dtEVs with an integrated membrane exhibit resistance to degradation. We performed RNase A treatment (ThermoFisher, EN0531, 5 µg/mL, 30 min at 37°C) with or without permeabilization of the dtEV membrane using 0.3% Triton X-100 (Sigma-Aldrich). By evaluating the loading of TP53 mRNA **a,** and siKRAS^{G12D} **b,** in dtEVs, we observed a significant reduction in both TP53 mRNA and siKRAS^{G12D} content when conducting RNase treatment accompanied by Triton X-100 permeabilization [TP53 mRNA: Before: 3.06±0.83; RNase: 2.44±0.33 RNase + Triton: 7.14±0.11x 10^{-5}; siKRASG^{12D}: 1147.0±217.7; 1086.0±196.4; 86.3±48.11, n=3]. Data is presented as mean ± SD over three biological independent experiments.

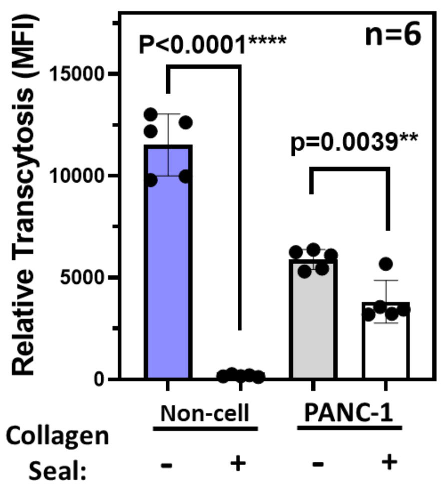

Supplementary Figure 30 | Collagen sealing in transcytosis assay prevented the leakage of bypassed dtEVs. EVs (1x10^{6} per PANC-1 cell) were first labeled by PKH67 (Sigma-Aldrich). The PANC-1 cells were seeded on the porous membrane surface of a Transwell® insert as monolayer. Once the PANC-1 cells reached 90% confluence, we applied a 1% collagen solution (C5533, Sigma-Aldrich) for 1 h to seal potential leakage between the cells and the Transwell® before conducting the transcytosis assay. To assess the occurrence of leakage, we compared the dtEVs that bypassed the non-cell control (only porous Transwell®) or PANC-1 cells with or without collagen sealing. The collagen sealing significantly prevented the leakage of bypassed dtEVs (MFI of by passed dtEVs: Non-cell: 11519±1523; Non-cell sealed: 184.8±55.64; PANC-1: 5899±489.9; PANC-1 sealed: 3821±1048). Data is presented as mean ± SD over six biological independent experiments.

Supplementary Figure 31 | Uncropped blots.

Scan of Fig. 1d

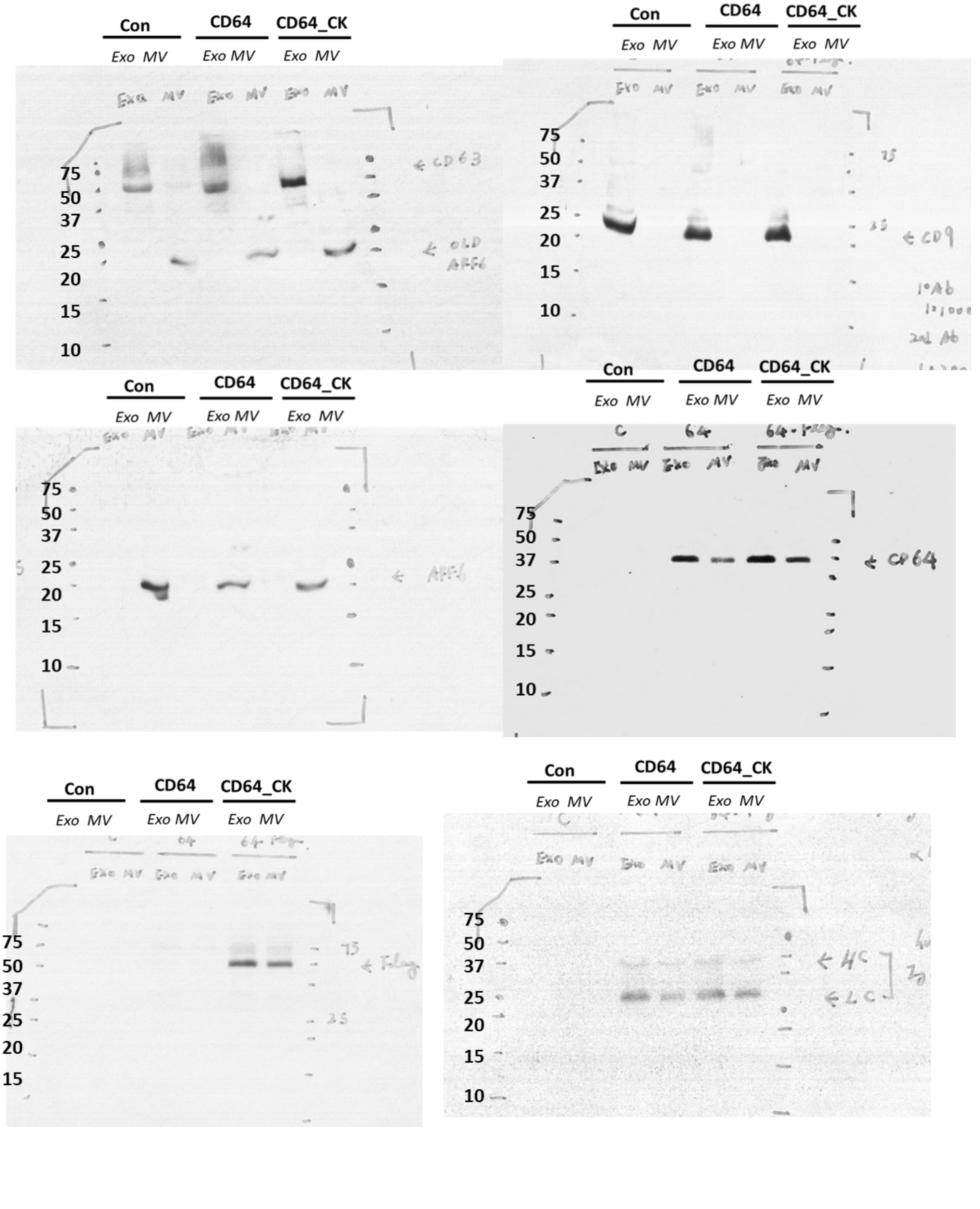

Scan of Fig. 2d

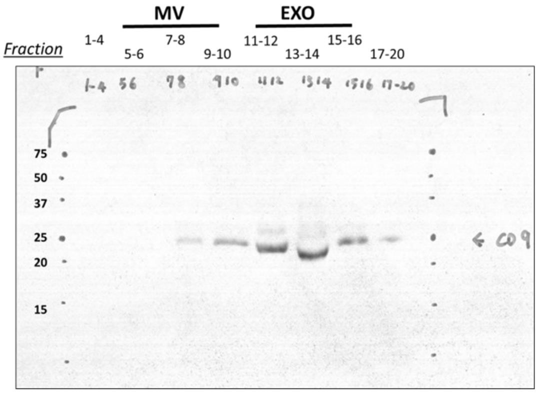

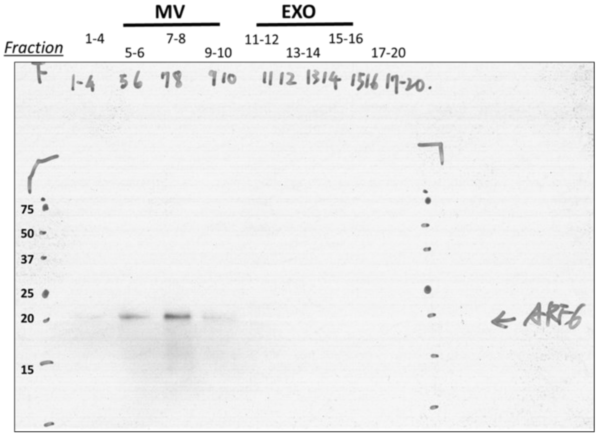

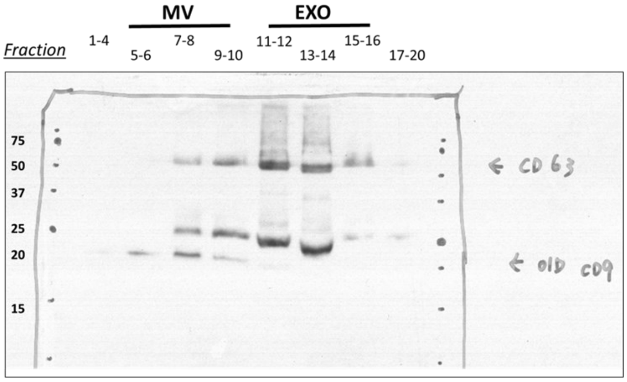

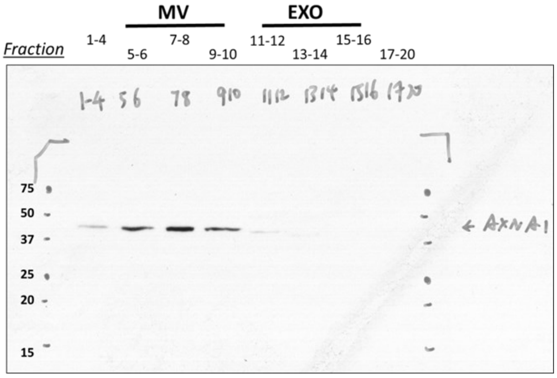

Scan of Fig. 5b

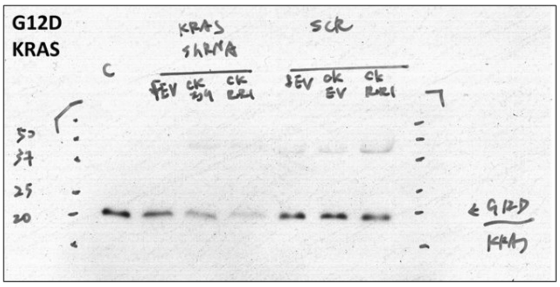

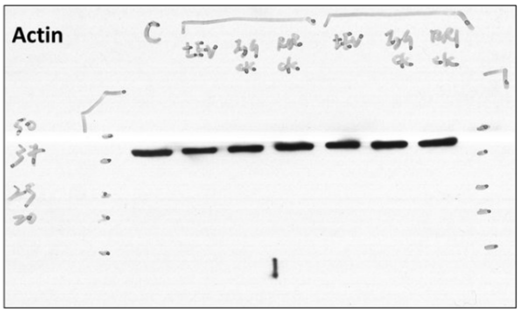

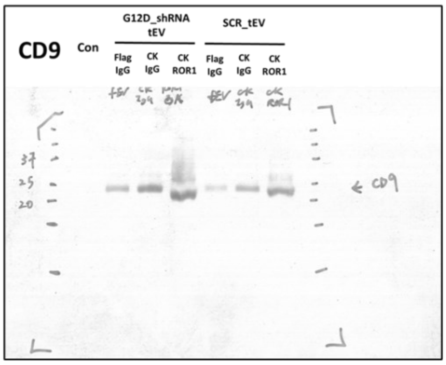

Scan of Fig. 5c

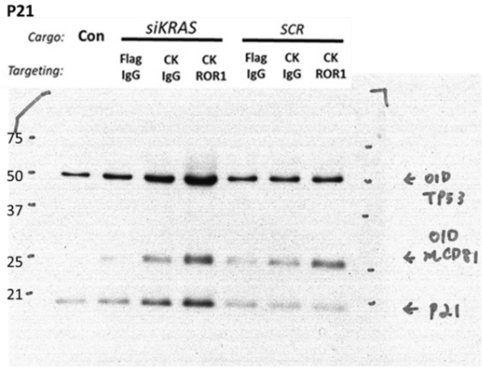

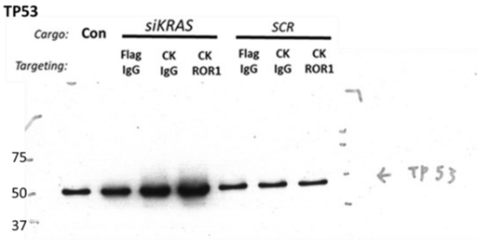

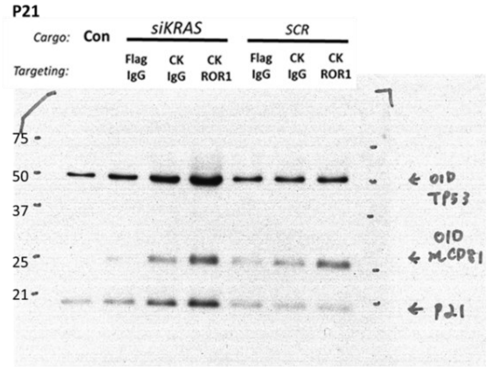

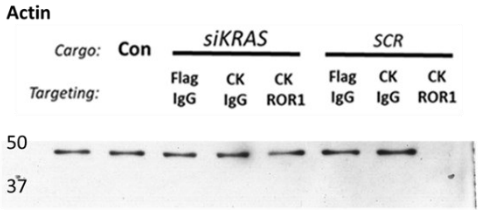

Scan of Fig. 5d

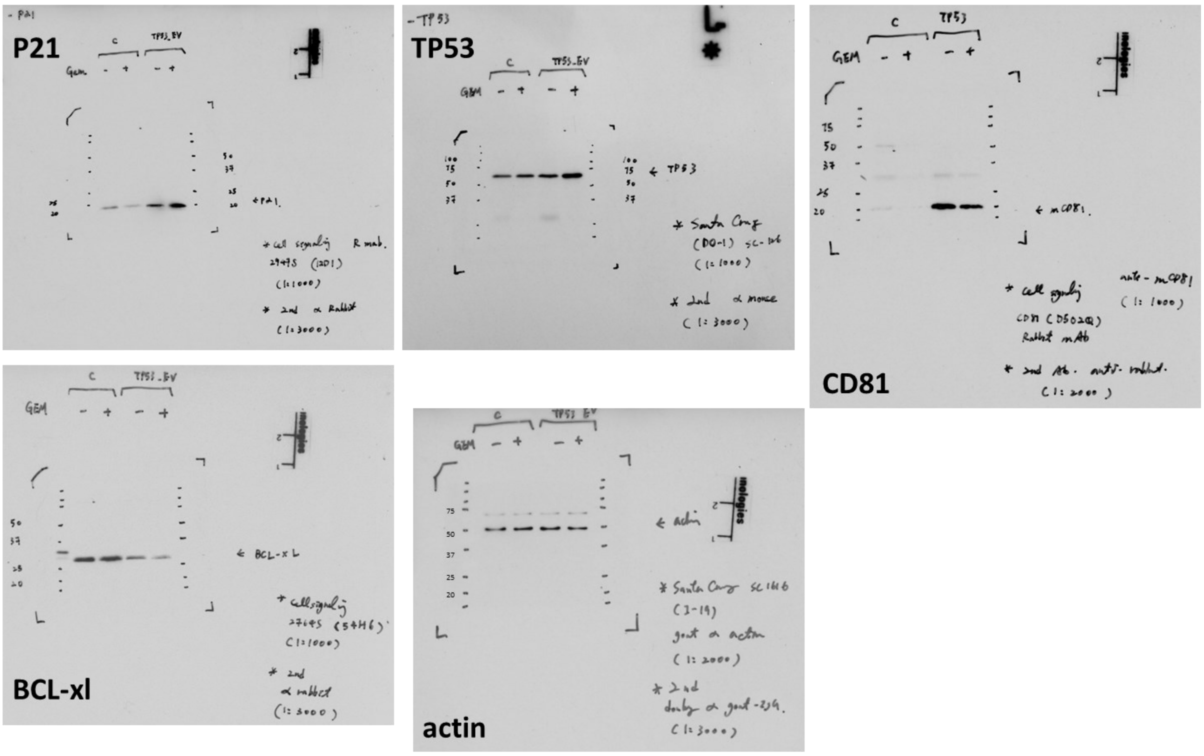

Scan of Fig. 5e

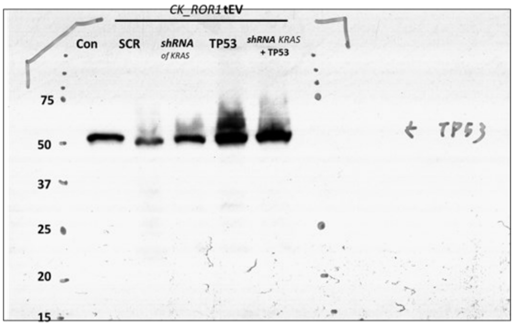

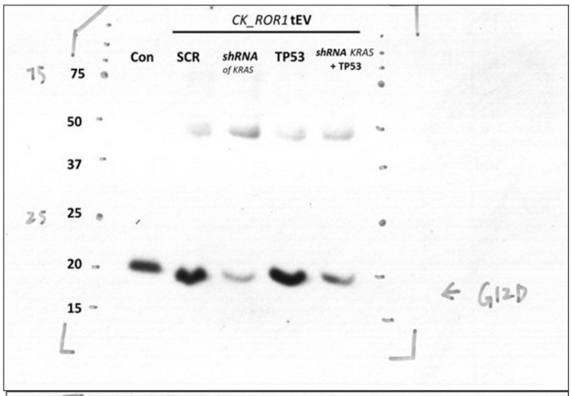

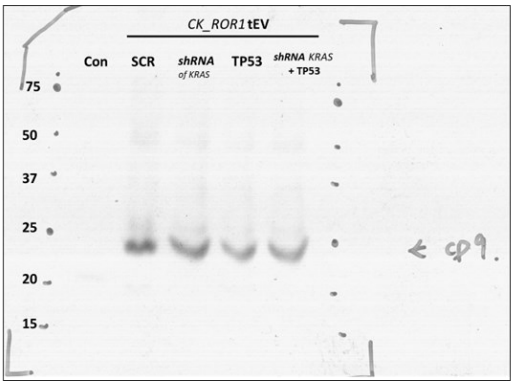

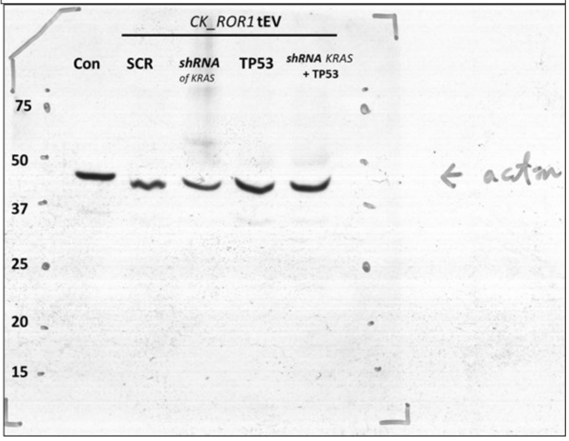

Scan of Fig. S19

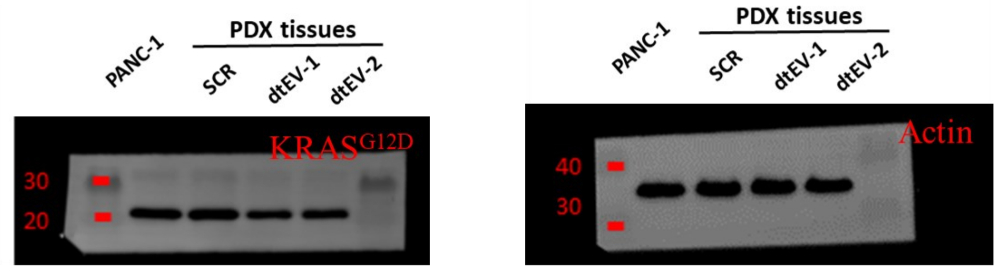

Scan of Fig. S21

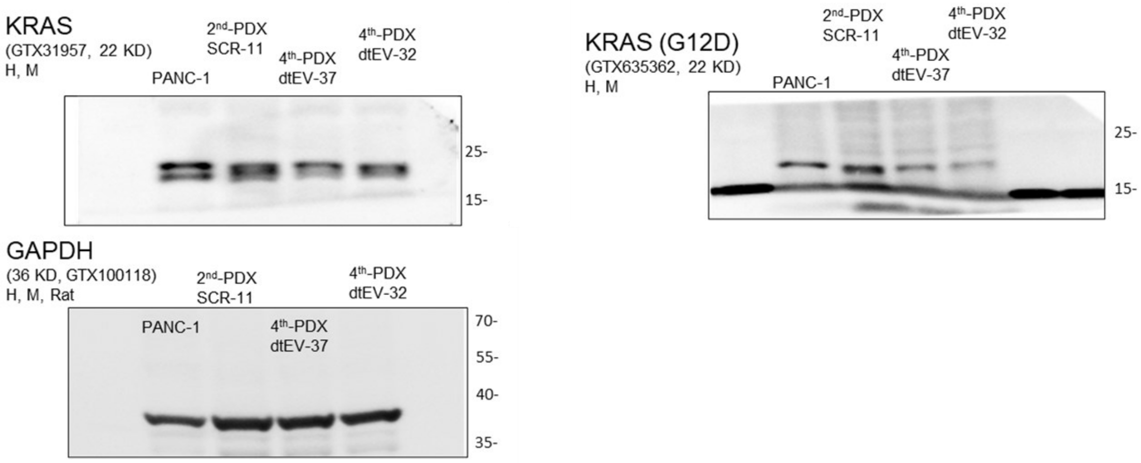

Supplementary References

1. Nguyen LTH*, et al.* An immunogold single extracellular vesicular RNA and protein ((Au) SERP) biochip to predict responses to immunotherapy in non-small cell lung cancer patients. *J Extracell Vesicles* **11**, e12258 (2022).
