# Supplementary material for: Dual targeted extracellular vesicles regulate oncogenic genes in advanced pancreatic cancer
Source: Nat Commun. 2023 Oct 23;14:6692. doi: 10.1038/s41467-023-42402-3 (PMC10593751; doi:10.1038/s41467-023-42402-3)
Supplement: Supplementary file 3 — Description of Additional Supplementary Files [file 41467_2023_42402_MOESM3_ESM.pdf]

Description of Additional Supplementary Files

Supplementary Data 1 | Changes of EV microRNAs from TACE stimulated hBMSCs with or without TP53 plasmid. More details can be found in GEO accession GSE223409.

Supplementary Data 2 | Changes of EV microRNAs from TACE stimulated MEFs with or without TP53 plasmid. More details can be found in GEO accession GSE223409.
