# Supplementary material for: Dual targeted extracellular vesicles regulate oncogenic genes in advanced pancreatic cancer
Source: Nat Commun. 2023 Oct 23;14:6692. doi: 10.1038/s41467-023-42402-3 (PMC10593751; doi:10.1038/s41467-023-42402-3)
Supplement: Supplementary file 6 — Reporting Summary [file 41467_2023_42402_MOESM6_ESM.pdf]

nature portfolio

Corresponding author(s): L. James Lee
NCOMMS-23-18300

Last updated by author(s): Oct 4, 2023

Reporting Summary

Nature Portfolio wishes to improve the reproducibility of the work that we publish. This form provides structure for consistency and transparency in reporting. For further information on Nature Portfolio policies, see our Editorial Policies and the Editorial Policy Checklist.

Statistics

For all statistical analyses, confirm that the following items are present in the figure legend, table legend, main text, or Methods section.

n/a	Confirmed	
☐	☒	The exact sample size (*n*) for each experimental group/condition, given as a discrete number and unit of measurement
☐	☒	A statement on whether measurements were taken from distinct samples or whether the same sample was measured repeatedly
☐	☒	The statistical test(s) used AND whether they are one- or two-sided<br>*Only common tests should be described solely by name; describe more complex techniques in the Methods section.*
☐	☒	A description of all covariates tested
☐	☒	A description of any assumptions or corrections, such as tests of normality and adjustment for multiple comparisons
☐	☒	A full description of the statistical parameters including central tendency (e.g. means) or other basic estimates (e.g. regression coefficient) AND variation (e.g. standard deviation) or associated estimates of uncertainty (e.g. confidence intervals)
☐	☒	For null hypothesis testing, the test statistic (e.g. F, t, r) with confidence intervals, effect sizes, degrees of freedom and P value noted<br>*Give P values as exact values whenever suitable.*
☒	☐	For Bayesian analysis, information on the choice of priors and Markov chain Monte Carlo settings
☒	☐	For hierarchical and complex designs, identification of the appropriate level for tests and full reporting of outcomes
☒	☐	Estimates of effect sizes (e.g. Cohen's *d*, Pearson's *r*), indicating how they were calculated

Our web collection on statistics for biologists contains articles on many of the points above.

Software and code

Policy information about availability of computer code

Data collection	TIRF microscopy (Nikon Eclipse Ti Inverted Microscope System) was used to acquire images. Images were collected by an Andor iXon EMCCD camera with an Â~100 lens. MATLAB software (R2019B) and Image J (1.8.0) were used to analyze the images.
Data analysis	The custom MATLAB code used to process the imaging data analysis. All statistical data were analyzed with Prism 7 Graph Pad, Minitab 18, Original 2019 and Excel.

For manuscripts utilizing custom algorithms or software that are central to the research but not yet described in published literature, software must be made available to editors and reviewers. We strongly encourage code deposition in a community repository (e.g. GitHub). See the Nature Portfolio guidelines for submitting code & software for further information.

Data

Policy information about availability of data

All manuscripts must include a data availability statement. This statement should provide the following information, where applicable:

- Accession codes, unique identifiers, or web links for publicly available datasets
- A description of any restrictions on data availability
- For clinical datasets or third party data, please ensure that the statement adheres to our policy

The non-coding RNA profiling data in this study are available in the GEO database under the accession code GSE223409. All other data supporting the findings of

this study are available within the article and its supplementary files. Any additional requests for information can be directed to the corresponding author (L.J.L.). Source data are provided with this paper.

Human research participants

Policy information about studies involving human research participants and Sex and Gender in Research.

Reporting on sex and gender	N/A
Population characteristics	The study was limited to tumor cells samples from patients with pancreatic cancer. Genotyping and characteristics of samples from different hospitals are described Methods section.
Recruitment	1. Fresh surgical specimen was obtained from a male patient diagnosed with PDAC (termed T26), who underwent pancreaticoduodenectomy at National Cheng Kung University Hospital (NCKUH) in Taiwan. The study was approved by the Institutional Review Board (IRB number: A-ER-106-157) of NCKUH, and informed consent was ob¬tained from the patient.<br>2. Fresh surgical specimen was obtained from a patient diagnosed with PDAC (Patient No. PA6247, Crown Bioscience Inc, China). The IRB protocol was approved to collected human sample and informed consent waived.
Ethics oversight	All patients in the study consented to have their samples used in the study, under IRB approval.

Note that full information on the approval of the study protocol must also be provided in the manuscript.

Field-specific reporting

Please select the one below that is the best fit for your research. If you are not sure, read the appropriate sections before making your selection.

☒ Life sciences ☐ Behavioural & social sciences ☐ Ecological, evolutionary & environmental sciences

For a reference copy of the document with all sections, see nature.com/documents/nr-reporting-summary-flat.pdf

Life sciences study design

All studies must disclose on these points even when the disclosure is negative.

Sample size	No statistical methods were used to predetermine sample sizes, which for each retrospective cohort were determined by the number of patients with sample available.
Data exclusions	Following the IACUC protocol, experimental mice had to be removed as reaching Expected Early Removal Criteria (ERC). In the orthotic PDX experiment (Fig. 6h), several engrafted mice after abdominal surgery were removed once reaching the ERC condition as tumor burden >30% body wight, or 20% loss of body weight, respiratory distress, mouse body condition score <2 (from 5), or very large abdominal organs that impede mobility. In orthotopic PDX experiment (Fig. 6h), one mouse was removed from dtEVGli-1 group because of ERC condition. No mice in the overall survival were excluded from the data analysis.
Replication	Information on experimental replication is provided in Methods and in figure legends. Replicate numbers were chosen based on the minimum number required to detect differences between groups in all cell-culture experiments. Number of mice in each treatment in animal studies were considered based on achievable patient samples and mice.
Randomization	Recipient mice in each study cohort were first engrafted with PDAC cancer cells (PANC-1 cell line or PDX), and then weekly monitored for the tumor growth. Only the engrafted mice were randomized and assigned for further treatment. All available samples from these individual mice were analyzed as described.
Blinding	The investigators were not blinded for the sample analysis from animal experiments.

Reporting for specific materials, systems and methods

We require information from authors about some types of materials, experimental systems and methods used in many studies. Here, indicate whether each material, system or method listed is relevant to your study. If you are not sure if a list item applies to your research, read the appropriate section before selecting a response.

Materials & experimental systems

n/a	Involved in the study	
☐	☒	Antibodies
☐	☒	Eukaryotic cell lines
☒	☐	Palaeontology and archaeology
☐	☒	Animals and other organisms
☒	☐	Clinical data
☒	☐	Dual use research of concern

Methods

n/a	Involved in the study	
☒	☐	ChIP-seq
☐	☒	Flow cytometry
☒	☐	MRI-based neuroimaging

Antibodies

Antibodies used

Targeted antibodies (No carriers): anti-ROR1 (humanized, 2A2, Creative Biolabs, NY, USA), anti-EGFR (humanized, Hu1, R&D systems, MN, USA)
Pure antibodies: anti-ARF6 antibody (NBP2-41263, Novus Biologicals, MN, USA), anti-Annexin A1 antibody (AF3770, R&D systems, MN, USA), anti-KRAS_G12D (D8H7, Cell Signaling Technology, MA, USA), anti-TP53 (DO-1, sc-126, Santa Cruz Biotechnology, TX, USA), anti-CD64 (10.1, sc-1184, Santa Cruz Biotechnology, TX, USA), anti-hIgG (A18847, ThermoFisher Scientific, MA USA)
anti-actin (I-19, sc-1616, Santa Cruz Biotechnology, TX, USA), anti-Annexin A1 antibody (AF3770, R&D systems, MN, USA), anti-MCOLN1/TRPML1 (Polyclonal, PA1-46474, ThermoFisher Scientific, MA USA), anti-HSP40 (C64B4, Cell Signaling Technology, MA, USA), anti-HSP70 (3A3, sc-32239, Santa Cruz Biotechnology, TX, USA), anti-flag (PA1-984B-HRP, ThermoFisher Scientific, MA USA), anti-p21 (12D1, Cell Signaling Technology, MA, USA), anti-BCL-xl (H-5, sc-8392, Santa Cruz Biotechnology, TX, USA), anti-CD63 (sc-5275, Santa Cruz Biotechnology, TX, USA), anti-CD9 -Alexa Fl (sc-13118 AF546, Santa Cruz Biotechnology, TX, USA), anti-CD81 (sc-166029 AF647, Santa Cruz Biotechnology, TX, USA), anti-Rab5 (C8B1, Cell Signaling Technology, MA, USA), anti-Rab7 (D95F2, Cell Signaling Technology, MA, USA), LAMP-1 Antibody (H4A3, sc-20011, Santa Cruz Biotechnology, TX, USA), LAMP-2 Antibody (H4B4, sc-18822, Santa Cruz Biotechnology, TX, USA), anti-Ki67 (ab15580, Abcam, MA, USA), and Recombinant Anti-Ras _mutated G12D (HL10, ab289373, Abcam, MA, USA).
Conjugated Antibodies: Biotin anti-CD63 antibody (ab134331, Abcam, MA, USA), Biotin anti-CD9 antibody (ab28094, Abcam, MA, USA), Biotin anti-CD81 antibody (ab239238, Abcam, MA, USA), FITC-conjugated human IgG (from human serum, F9636, Millipore Sigma, MA, USA), PE-conjugated anti-CD64 (10.1, 12-0649-42, ThermoFisher Scientific, MA USA), APC-conjugated anti-CD64 (10.1, 17-0649-42, ThermoFisher Scientific, MA USA), Alexa Fluor® 488-conjugated anti-ROR1 (FAB2000G, R&D systems, MN, USA), and PE-conjugated anti-EGFR (MA5-28544, ThermoFisher Scientific, MA USA)

Validation

All antibodies were purchased commercially and were validated by the manufacturers.

Eukaryotic cell lines

Policy information about cell lines and Sex and Gender in Research

Cell line source(s)

Mouse embryonic fibroblasts (MEFs) were purchased from MilliporeSigma (MA, USA).
PANC-1 cell line were from American Type Culture Collection (ATCC, USA).
Human bone marrow-derived mesenchymal stem cells (hBMSCs) were purchased from RoosterBio (MD, USA)

Authentication

MEF cells were authenticated by MilliporeSigma.
PANC-1 cells were authenticated by ATCC.
hBMSCs were authenticated from RoosterBio.

Mycoplasma contamination

All cell lines tested negative for mycoplasma contamination.

Commonly misidentified lines
(See ICLAC register)

No commonly misidentified cell lines were used.

Animals and other research organisms

Policy information about studies involving animals; ARRIVE guidelines recommended for reporting animal research, and Sex and Gender in Research

Laboratory animals

NOD/SCID mice were purchased from Shanghai Slack Laboratory Animals in China, National Laboratory Animal Center, or Laboratory Animal Center at Adademia Sinica in Taiwan. BALB/c-Nude mice were purchased from Shanghai Slack Laboratory Animals in China.

Wild animals

N/A

Reporting on sex

6~8-week-old male NOD/SCID or BALB/c-Nude mice were used for the xenoengraft in animal experiments.

Field-collected samples

All mice were kept and operated at a housing temperature of 20-25 °C following the Guidelines of the Institutional Animal Care and Use Committee (IACUC). The immunodeficient (NOD/SCID and BALB/c-Nude) mice were maintained in microisolator (filter bonneted), pressurized, individually ventilated cages, and the food, water, bedding, and cages were sterilized. The animal surgeries were operated in the laminar flow hood. The weekly imaging of mice was taken by IVIS® Spectrum In Vivo Imaging System (PerkinElmer, MA, USA) and Inveon Micro-PET/CT system (Siemens, Germany).

Ethics oversight

All animal experiments were approved by the Animal Ethics Committee and experimental procedures were conducted in accordance with the Guidelines of the Institutional Animal Care and Use Committee (IACUC).
NOD/SCID mice were purchased from Shanghai Slack Laboratory Animals in China (IACUC: 2021-0019, approved by Shanghai Model Organisms Center Inc.) or Laboratory Animal Center at Academia Sinica in Taiwan (AS-IACUC-18-03).

Note that full information on the approval of the study protocol must also be provided in the manuscript.

Flow Cytometry

Plots

Confirm that:

☒ The axis labels state the marker and fluorochrome used (e.g. CD4-FITC).

☒ The axis scales are clearly visible. Include numbers along axes only for bottom left plot of group (a 'group' is an analysis of identical markers).

☒ All plots are contour plots with outliers or pseudocolor plots.

☒ A numerical value for number of cells or percentage (with statistics) is provided.

Methodology

Sample preparation	PANC-1 cells treated with EVs for 24h were collected and stained with FITC Annexin V Apoptosis/PI Detection Kit. For cell cycle assay, the collected cells were first fixed and permeabilized by cold 70% ethanol for 30 min and treated with ribonuclease (20 μg/ml) for 30 min. The permeabilized cells were stained propidium iodide (50 μg/ml) overnight at °C
Instrument	Gallios Flow Cytometry (3 Laser/ 10 Color Detection), Beckman Coulter, CA, USA
Software	Kaluza, Beckman Coulter, CA, USA
Cell population abundance	Over 10,000 events were captured by a Flow cytometry for analysis.
Gating strategy	The cell population was gated by singlet selection through FSC-H(height) versus FSC-A (area) to cut off doublets or debris. The fluorescent signal was determined on singlet population.

☒ Tick this box to confirm that a figure exemplifying the gating strategy is provided in the Supplementary Information.
